# Antioxidant and Anticancer Potential of Bioactive Compounds from *Rhinacanthus nasutus* Cell Suspension Culture

**DOI:** 10.3390/plants11151994

**Published:** 2022-07-30

**Authors:** Pattralak Songserm, Poramaporn Klanrit, Poramate Klanrit, Jutarop Phetcharaburanin, Pornthap Thanonkeo, Jirawan Apiraksakorn, Khamphee Phomphrai, Preekamol Klanrit

**Affiliations:** 1 Graduate School, Khon Kaen University, Khon Kaen 40002, Thailand; pattralaks@kkumail.com; 2 Department of Biotechnology, Faculty of Technology, Khon Kaen University, Khon Kaen 40002, Thailand; portha@kku.ac.th (P.T.); jirapi@kku.ac.th (J.A.); 3 Research Group of Chronic Inflammatory Oral Diseases and Systemic Diseases Associated with Oral Health, Department of Oral Biomedical Sciences, Faculty of Dentistry, Khon Kaen University, Khon Kaen 40002, Thailand; porakla@kku.ac.th; 4 Department of Biochemistry, Faculty of Medicine, Khon Kaen University, Khon Kaen 40002, Thailand; porakl@kku.ac.th (P.K.); jutarop@kku.ac.th (J.P.); 5 Cholangiocarcinoma Research Institute, Faculty of Medicine, Khon Kaen University, Khon Kaen 40002, Thailand; 6 Fermentation Research Center for Value Added Agricultural Products (FerVAAP), Khon Kaen University, Khon Kaen 40002, Thailand; 7 School of Molecular Science and Engineering, Vidyasirimedhi Institute of Science and Technology, Rayong 21210, Thailand; khamphee.p@vistec.ac.th

**Keywords:** anticancer, antioxidant, callus, cell suspension culture, *Rhinacanthus nasutus*

## Abstract

The potential benefits of natural plant extracts have received attention in recent years, encouraging the development of natural products that effectively treat various diseases. This is the first report on establishing callus and cell suspension cultures of *Rhinacanthus nasutus* (L.) Kurz. A yellow friable callus was successfully induced from in vitro leaf explants on Murashige and Skoog medium supplemented with 1 mg/L 2,4-dichlorophenoxyacetic acid and 1 mg/L 1-naphthalene acetic acid. A selected friable callus line was used to establish the cell suspension culture with the same medium. The antioxidant assays showed that the leaf- and ethanolic-suspension-cultured cell (SCC) extracts exhibited high antioxidant potential. In addition, the in vitro cytotoxicity revealed by the MTT assay demonstrated potent antiproliferative effects against the oral cancer cell lines ORL-48 and ORL-136 in a dose-dependent manner. Several groups of compounds, including terpenoids, phenolics, flavonoids, quinones, and stilbenes, were identified by UHPLC–QToF–MS, with the same compounds detected in leaf and SCC extracts, including austroinulin, lucidenic acid, esculetin, embelin, and quercetin 3-(2″-p-hydroxybenzoyl-4″-p-coumarylrhamnoside). The present study suggests the value of further investigations for phytochemical production using *R. nasutus* cell suspension culture.

## 1. Introduction

*Rhinacanthus nasutus* (L.) Kurz (*R. nasutus*), also known as snake jasmine or Thong Pan Chang (in Thai), is a high-value medicinal and versatile plant that belongs to the Acanthaceae family. It is widely cultivated in tropical and subtropical areas, including India, China, and Thailand [1]. Different parts of *R. nasutus*, including the leaves and roots, have also been used in traditional medicine for the treatment of various diseases and disorders, including skin diseases such as eczema, herpes, inflammation, hepatitis, diabetes, and hypertension, as well as bacterial, fungal, or viral infections [2,3,4]. *R. nasutus* has been shown to contain significant levels of essential bioactive compounds; for example, phenolic compounds, including phenolic acids, stilbenes, lignans, and flavonoids, as well as other groups of compounds, including steroids, terpenoids, and quinones, which have been isolated from and characterized in the leaves and roots [4,5,6,7]. These bioactive compounds offer several attractive biological properties and therapeutic potential, such as antibacterial, antifungal, antiviral, antioxidant, neuroprotective, and anticancer effects, making *R. nasutus* the subject of research in several fields [7,8,9,10,11,12].

Most of the relevant research about *R. nasutus* has mainly focused on extracting bioactive compounds from plant parts, identifying the bioactive compounds, and investigating their potential biological properties. However, the main drawback of using plant parts from nature or traditional cultivation is the potential for many diseases and pathogens, decreasing their survival and leading to contamination. Moreover, the accumulation levels of bioactive compounds vary depending on the geography and environmental influences. Thus, plant tissue and cell cultures are alternative techniques used to circumvent these problems. Compared with traditional growth, plant tissue cultures offer several advantages, including the rapid multiplication of plant species in a short period, the production of pathogen-free plants, and the continuous production of commercially important bioactive compounds using a callus or cell suspension culture.

Plant callus and cell suspension cultures exhibit superior secondary metabolite production over an extended subculture period compared to extraction from natural plant leaves [13]. The induction of leaf-derived callus and cell suspension cultures is an alternative technique for inducing the same group of bioactive compounds present in the leaf. The callus induction is the first step in the establishment of cell suspension cultures, in which friable callus fragments are placed into suitable sterile liquid media [14]. The types and concentrations of plant growth regulators (PGRs) used in the system are essential parameters for successful callus induction. The fine cell suspension culture provides an excellent opportunity to investigate the properties and potentialities of plant cells. Moreover, the cell suspension culture offers a valuable platform for producing high-value secondary metabolites and other invaluable bioactive compounds of commercial interest [15,16].

Several studies have demonstrated the in vitro production of secondary metabolites from many plants for various purposes. For example, the roots of *Panax ginseng* are used for the production of ginsenoside and hypocotyls of *Celosia argentea* are used as explants for the production of betalains as color compounds [17,18]. To date, many studies on the micropropagation of *R. nasutus* to generate new whole plants using various explant techniques have been published [19,20]. However, only a few studies have reported on the leaf-derived callus induction of *R. nasutus* without further establishment for bioactive compound production [21,22,23]. For callus induction, it was found that using Murashige and Skoog (MS) medium supplemented with different concentrations of PGRs, such as 5 mg/L 6-benzylaminopurine (BAP) and 2.5 mg/L 2,4-dichlorophenoxyacetic acid (2,4-D) [21], 0.1 mg/L thidiazuron (TDZ) and 0.5 mg/L 2,4-D [24], and 4 mg/L kinetin (KT) and 0.5 mg/L indole-3-butyric acid (IBA) [23] or 2 mg/L 2,4-D alone, was successful in generating different types of callus [21,22]. However, the callus texture was compact and was not suitable for establishing a cell suspension culture system. Moreover, the study of bioactive compound production using suspension culture and the potential of the suspension-cultured cell (SCC) extract from *R. nasutus* have not been investigated.

The aims of this work were to establish callus and cell suspension cultures of *R. nasutus* and to investigate the antioxidant and anticancer potentials of the extracts. In this study, leaf explants were used for callus induction with different PGRs at various concentrations. Several parameters were determined, including the percentages of callus induction, callus appearance, and the growth profile of the cell suspension culture. In addition, the metabolite profiles of leaf and SCC extracts of *R. nasutus* were explored using ultra high performance liquid chromatography–quadrupole time-of-flight mass spectrometry (UHPLC–QToF –MS).

## 2. Results

### 2.1. Callus Induction and Proliferation

**Figure 1:**
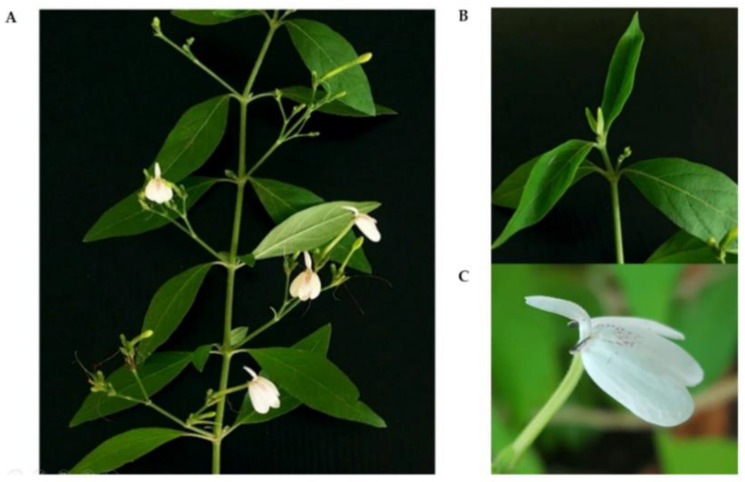
*Rhinacanthus nasutus* (L.) Kurz—A Thai medicinal plant used in the present study: (**A**) a mature plant, (**B**) a shoot tip, and (**C**) a flower.

*R. nasutus* is a small shrub with a height of 50–200 cm. The leaves are simple, opposite, and ovate to oblong. The flowers contain two upper lips and three lower lips (Figure 1). After sterile *R. nasutus* plants were established from shoot tips, the young leaf segments developed from 6-week-old shoots were used as explants for the callus induction experiment. The leaf explants were cultured on MS medium supplemented with different concentrations of 2,4-D and 1-naphthalene acetic acid (NAA) (Table 1).

**Figure 2:**
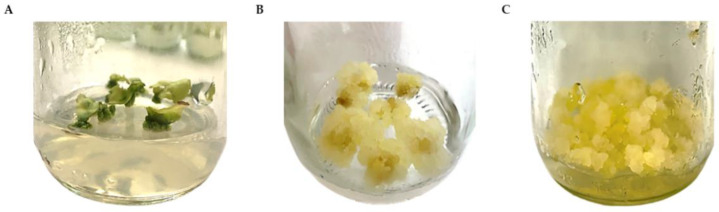
Callus culture of *R. nasutus*: (**A**) callus initiation from leaf explants after 3 weeks; (**B**) callus growth on CIM (MS medium supplemented with 1 mg/L 2,4-D and 1 mg/L NAA) after culturing for 7 days; (**C**) callus proliferation on CIM after 15 days of culture.

The callus was initiated from the cut edges of leaf explants, and the callus formation was initiated within 3 weeks in almost all treatments (Figure 2A). The combination of 2,4-D (0.5–2.0 mg/L) and NAA (1.0 and 2.0 mg/L) was found to be effective for callus induction. The maximum callogenesis response of 100% was observed in the presence of 2,4-D (0.5, 1, 1.5, and 2 mg/L) and 1.0 mg/L NAA. In addition, the induced callus was yellowish and friable in texture (Figure 2B). The supplementation of the medium with 2 mg/L NAA resulted in lower percentages of callus induction (<35%), and using either 2,4-D or NAA alone did not promote new callus growth (Table 1). No callus developed on the explants placed on the MS medium without PGRs, as expected. After five weeks, the callus derived from leaf explants was subcultured for proliferation on fresh MS medium supplemented with 1.0 mg/L 2,4-D and 1.0 mg/L NAA (callus induction medium, CIM) every two weeks for twenty-one subcultures. The yellow friable callus proliferated well and appeared as bright yellow and soft (Figure 2C).

### 2.2. Establishment of Cell Suspension Culture

**Figure 3:**
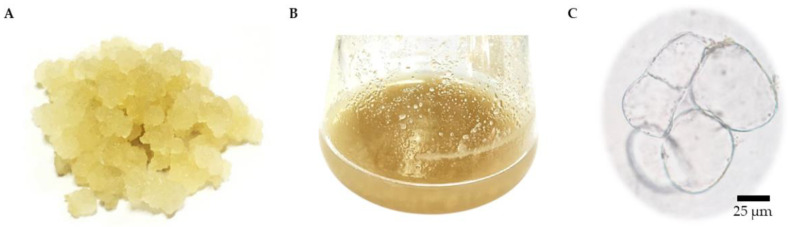
Establishment of *R. nasutus* cell suspension culture: (**A**) leaf-derived yellow friable callus; (**B**) SCCs of *R. nasutus*; (**C**) morphology of 15 day friable callus under a bright-field microscope. The scale bar represents 25 µm.

**Figure 4:**
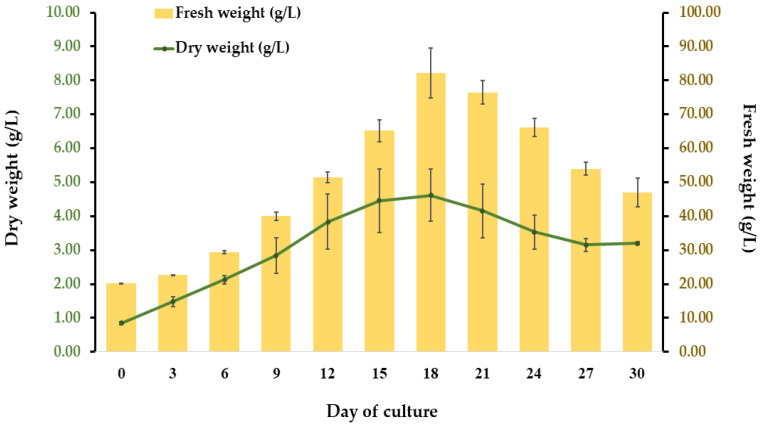
Growth profile of *R. nasutus* cell suspension cultures during 30 days of culture in liquid CIM.

A fast-growing cell line producing a yellow friable callus was used to initiate a cell suspension culture (Figure 3A). SCCs were grown in liquid CIM, i.e., MS medium supplemented with 1 mg/L 2,4-D and 1 mg/L NAA (Figure 3B). Homogeneous cell masses were observed under a bright-field microscope with Evans blue staining, showing oval and spherical cells with thin walls (Figure 3C). The growth pattern of the *R. nasutus* suspension culture was typical of plant cell cultures, with a fresh weight (FW) pattern similar to the dry weight (DW) pattern. The cells continued to grow until they reached maximum biomass accumulation on day 18, when FW and DW values of 82.14 g/L and 4.62 g/L, respectively, were observed (Figure 4). After this, the biomass decreased.

To determine the most appropriate time point to collect SCCs and further test for their antioxidant and anticancer potential, the total phenolic content (TPC) was used as a marker to ensure that the obtained fast-growing cell line actually produced bioactive compounds. The SCCs were collected at three time points, namely days 9, 15, and 18, and were tested for TPC values. The SCCs showed the highest TPC on day 15, with a value of 14.67 ± 0.37 mg GAE/g DW (Figure S1). The SCCs observed on day 9 and day 15 were viable, as demonstrated by the callus color and texture, as well as the images viewed under a bright-field microscope with Evans blue staining. However, the SCCs observed on day 18 were mostly nonviable. Based on these results, the cell line culture for 15 days was used to prepare the crude extracts of the SCCs and to further investigate their antioxidant and anticancer properties compared to those of the leaf extract.

### 2.3. Total Phenolic and Total Flavonoid Contents

The leaf and SCC samples were extracted with 80% (*v*/*v*) ethanol using the maceration extraction method at 65 °C for 6 h. The TPC and TFC values obtained from the extracts were analyzed using the Folin–Ciocalteu (FC) and aluminum chloride colorimetric assays, respectively. The TPC and TFC values of the ethanolic leaf extract were significantly higher than those of the SCCs. The leaf extract showed a TPC of 45.27 ± 1.34 mg GAE/g DW and a TFC of 8.67 ± 0.10 mg QE/g DW, whereas the SCC extract showed TPC and TFC values of 14.67 ± 0.09 mg GAE/g DW and 0.54 ± 0.06 mg QE/g DW, respectively.

### 2.4. Antioxidant Properties

**Figure 5:**
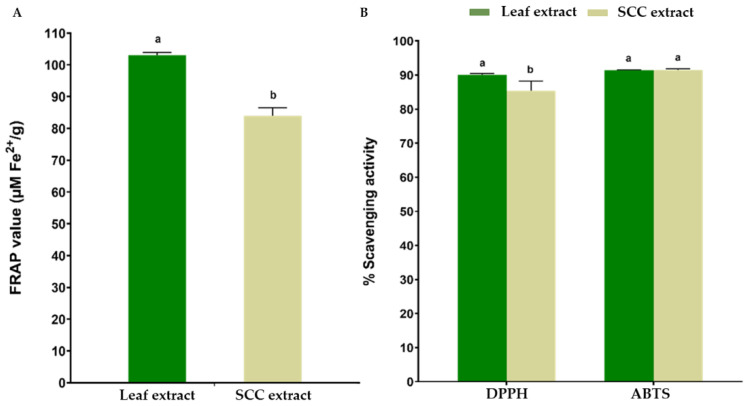
Antioxidant capacity of the ethanolic leaf and SCC extracts of *R. nasutus* (**A**) as determined by FRAP assay and (**B**) by DPPH and ABTS assays. The data are presented as the means ± SD of the results of triplicate determinations. Bars with different letters are significantly different according to an independent-samples *t* test at *p* ≤ 0.05.

The leaf and SCC extracts were tested for their antioxidant capacity using three methods, namely the ferric-reducing antioxidant power (FRAP), 2,2-diphenyl-1-picrylhydrazyl (DPPH), and 2,2′-azino-bis (3-ethylbenzothiazoline-6-sulfonic acid) (ABTS) assays (Figure 5). The FRAP value of the ethanolic leaf extract (103.05 ± 0.89 µM Fe^2+^/g DW) was higher than that of the SCC extract (84.01 ± 2.55 µM Fe^2+^/g DW) (Figure 5A). The DPPH scavenging activity of the leaf extract was 86.78%, which was higher than that of the SCC extract (83.29%). For the ABTS assay, the leaf and SCC extracts showed strong antioxidant capacity levels of 91.40% and 91.25%, respectively (Figure 5B).

### 2.5. Anticancer Activity

**Figure 6:**
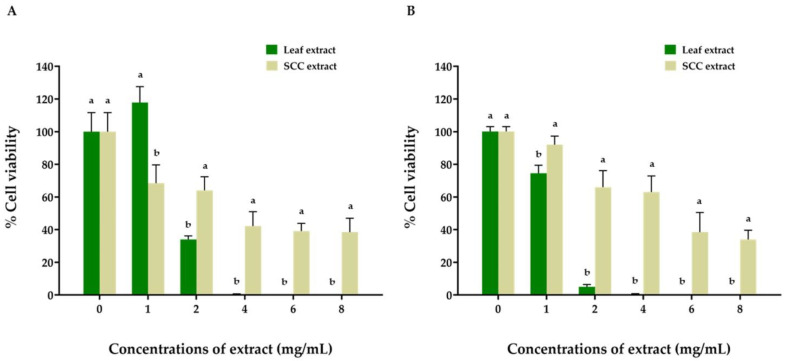
Oral cancer cell viability after treatment with ethanolic leaf and SCC extracts of *R. nasutus*: (**A**) ORL-48 cell line and (**B**) ORL-136 cell line. The data are presented as the means ± SD of the results of triplicate determinations. Bars with different letters at each concentration are significantly different according to an independent-samples *t* test at *p* ≤ 0.05.

The cell viability of the two ORL-136 and ORL-48 lines was assessed using the MTT assay following 48 h of treatment with the leaf and SCC extracts at concentrations of 1, 2, 4, 6, and 8 mg/mL. The results showed that both cell lines responded to the cytotoxic effects of both extracts in a dose-dependent manner based on the percentages of cancer cell viability (Figure 6). The leaf extract was shown to have a greater inhibitory effect on the growth of the ORL-48 (with an IC_50_ value of 2.35 mg/mL) and ORL-136 (with an IC_50_ of 1.24 mg/mL) cell lines than the SCC extract. In particular, after treatment with 4 mg/mL leaf extract, the results showed the significantly inhibited growth of the two cell lines, with a cell viability rate of 0%. In contrast, the SCC extract showed inhibitory effects on ORL-48 and ORL-136 cell growth at the highest concentration of 8 mg/mL, with cell viability levels of 38% and 34% and IC_50_ values of 4.38 mg/mL and 5.31 mg/mL, respectively.

The morphological analysis of the ORL-48 and ORL-136 cell lines, compared with the control group (no added extract), showed that the normal cancer cells were squamous and highly densely packed. No cancer cells survived in either cell line after treatment with 4 mg/mL leaf extract (Figure S2). Under treatment with the SCC extract at the highest dose of 8 mg/mL, some ORL-48 cancer cells survived; however, the shapes of the cells were abnormal, changing to spheroid with loose contact and more spaces between cells (Figure S3). The levels of cell death gradually increased in a dose-dependent manner. Similar results were observed for the ORL-136 cell line under the same treatment conditions.

### 2.6. Analysis of Metabolites in the Ethanolic Leaf and SCC Extracts by UHPLC–QToF–MS

The metabolite compositions of the leaf and SCC extracts were investigated using UHPLC–QToF–MS analysis in both positive and negative ionization modes and verified with public databases. Several groups of compounds were identified in the leaf and SCC extracts (Tables S1 and S2), belonging to 32 different classes (Table S3). High percentages of the secondary metabolites detected in both extracts were terpenoids or phenolic compounds, including phenolic acids, flavonoids, quinones, and stilbenes. Terpenoid compounds such as austroinulin, inulicin, lucidenic acid, and corosolic acid; phenolic compounds such as (E)-2-glucosyl-3,4′,5 trihydroxy stilbene, ferulic acid, trans-cinnamic acid, 4-hydroxycoumarin, and esculetin; quinones, including embelin, isoplumbagin, and 1,3,5,8-tetra hydroxy-6-methoxy-2-methyl anthraquinone; and flavonoids, including rutin and quercetin 3-(2″-p-hydroxybenzoyl-4″-p-coumarylrhamnoside), were observed in the ethanolic leaf extract (Table 2). The same groups of compounds were also detected in the SCC extract, with differences in compound types (Table 3). Interestingly, five compounds were observed in both the leaf and SCC extracts, namely, austroinulin, lucidenic acid, esculetin, embelin, and quercetin 3-(2″-p-hydroxybenzoyl-4″-p-coumarylrhamnoside) (Figure S4). The compounds present only in the SCC extract were camelledionol, pterostilbene glycinate, n-feruloyltyramine, 1,4-naphthoquinone, and hesperetin.

## 3. Discussion

Plant tissue and cell cultures, especially callus and cell suspension cultures, offer a promising platform for the production of high-value bioactive compounds from plants to be used for medical and pharmaceutical purposes [25,26,27]. PGRs, particularly auxins and cytokinins, play an important role in the growth and development of plant cells, and they have been widely used to induce callus formation in many plant species [27,28,29]. Calluses, unorganized, undifferentiated, and growing masses of cells formed by plant cell division, can be obtained from various types of explants placed on a solid medium containing either auxin alone or auxin together with cytokinin [30]. In the present investigation, a callus formation response of 100% using leaf explants of *R. nasutus* was achieved by using auxin alone (0.5–2 mg/L 2,4-D and 1 mg/L NAA), which was supplemented in MS medium. The leaf explant cultured on MS medium without any PGRs failed to produce a callus, as expected. Other studies have reported the use of only 2,4-D (2–5 mg/L) to induce a callus in *R. nasutus* [21,22]. However, our study showed no callus response when using 2,4-D alone at a concentration of 1 or 2 mg/L. This contradictory result could be attributed to the differences in the explant types used in the experiments, the different genetic backgrounds of the plants, and environmental influences [31]. In the case of using the combination of auxin and cytokinin for callus induction in *R. nasutus*, a few studies have demonstrated the use of 2,4-D together with either BAP or TDZ to induce callus formation. One study found that MS medium containing 0.1 mg/L 2,4-D and 0.5 mg/L TDZ was optimal for a high degree of callus induction (96%) [24], while another study demonstrated a callus induction frequency of 100% when culturing leaf explants on MS medium supplemented with 2.5 mg/L 2,4-D and 5 mg/L BAP [21]. In addition, other types of auxin and cytokinin, i.e., 0.5 mg/L IBA and 4 mg/L KT, respectively, were used to induce calluses, showing a callus induction rate of 98.8% [23].

**Table 2:** A list of metabolites in *R. nasutus* leaf extract.

Proposed Compound	Class *	Subclass *	Retention Time (min)	Product Ions (m/z)	Mass	Molecular Formula	Remarks
**Terpenoids**							
Austroinulin	Prenol lipids	Diterpenoids	43.58	345.24	322.25	C_20_ H_34_ O_3_	Found in *Stevia*/anti-inflammatory [32]
Inulicin	Prenol lipids	Terpene lactones	26.71	307.16	308.16	C_17_ H_24_ O_5_	May suppress angiogenesis and lung cancer cell growth [33]
Lucidenic acid	Prenol lipids	Sesquiterpenoids	41.91	481.26	458.27	C_27_ H_38_ O_6_	Lucidenic acid A, B, N caused cell cycle arrest in G_1_ phase/inhibited PMA-induced HCC invasion and apoptosis in human leukaemia cells HL-60 [34,35,36]
Corosolic acid	Prenol lipids	Triterpenoids	43.30	495.35	472.36	C_30_ H_48_ O_4_	Inhibits human colorectal cancer cells [37]/inhibits liver cancer cell growth under high-glucose conditions [38]
Ganoderic acid	Prenol lipids	Triterpenoids	40.42	555.29	532.31	C_30_ H_44_ O_8_	Inhibits cell proliferation, viability, and ROS and mRNA expression of TNF [39]
Punctaporin B	Prenol lipids	-	41.32	251.17	252.17	C_15_ H_24_ O_3_	Found in *Carolina cucumber* and *Vernonia cinerea* (L.) Less [40]
**Phenolics**							
(E)-2-glucosyl-3,4′,5 trihydroxy stilbene	Stilbenes	Stilbene glycosides	27.74	413.12	390.13	C_20_ H_22_ O_8_	Complementary and alternative medicine for cancer therapy [41]
Ferulic acid	Cinnamic acids and derivatives	Hydroxycinnamic acids and derivatives	16.07	193.05	194.06	C_10_ H_10_ O_4_	Able to suppress tumors in breast cancer, cervical carcinoma cells, prostate cancer cells, and pancreatic cancer cells [42]
Trans-cinnamic acid	Cinnamic acids and derivatives	Cinnamic acids	33.20	149.06	148.05	C_9_ H_8_ O_2_	Some inhibitory activity against enzymes from the human liver and the human cholangiocarcinoma cell line [43]
4-hydroxy coumarin	Coumarins and derivatives	Hydroxycoumarins	28.54	163.04	162.03	C_9_ H_6_ O_3_	Inhibits cell proliferation in the gastric carcinoma cell line [44]
Esculetin	Coumarins and derivatives	Hydroxycoumarins	13.12	177.02	178.03	C_9_ H_6_ O_4_	Inhibits migration and invasion of laryngeal cancer [45]
**Quinones**							
Embelin	Prenol lipids	Quinone and hydroquinone lipids	27.41	293.18	294.19	C_17_ H_26_ O_4_	Inhibits TNF-α and cancer cell metastasis [46,47,48]
Isoplumbagin	Naphthalenes	Naphthoquinones	23.39	187.04	188.05	C_11_ H_8_ O_3_	Suppresses oral, lung, prostate, and cervical cancer cells [49]
1,3,5,8-tetra hydroxy -6-methoxy-2-methyl anthraquinone	Others	Anthraquinones	28.85	334.09	316.06	C_16_ H_12_ O_7_	Inhibits cancer progression by kinases, topoisomerases, telomerases, matrix metallo proteinases, and G-quadruplexes [50]
**Flavonoids**							
2′,4′-dihydroxy-7-methoxy-8-prenylflavan	Flavonoids	Flavans	26.69	363.16	340.17	C_21_ H_24_ O_4_	Also found in *Cassia fistula* anticancer [51]
Rutin	Flavonoids	Flavonoid glycosides	14.92	609.15	610.15	C_27_ H_30_ O_16_	Anticancer effects on human cervical cancer cells [52]
Vitexin 4′-O-galactoside	Flavonoids	Flavonoid glycosides	15.52	593.15	594.16	C_27_ H_30_ O_15_	Many flavonoids have been isolated from and identified in flowers of the genus *Iris* [53]
Quercetin 3-(2′′-p-hydroxybenzoyl-4′′-p-coumaryl rhamnoside)	Flavonoids	Flavones	43.83	715.17	714.16	C_37_ H_30_ O_15_	Quercetin suppresses cyclooxygenase-2 (COX-2) expression in human breast cancer cells [54]

* Class and subclass were assigned according to the Human Metabolome Database (HMDB) (https://hmdb.ca) (accessed on 9 May 2022).

**Table 3:** A list of metabolites in *R. nasutus* SCC extract.

Proposed Compound	Class *	Subclass *	Retention Time (min)	Product Ions (m/z)	Mass	Molecular Formula	Remarks
**Terpenoids**							
Austroinulin	Prenol lipids	Diterpenoids	43.10	345.24	322.25	C_20_ H_34_ O_3_	Found in *Stevia*/anti-inflammatory [32]
Lucidenic acid	Prenol lipids	Sesquiterpenoids	38.46	463.31	462.30	C_27_ H_42_ O_6_	Lucidenic acid A, B, N caused cell cycle arrest in G_1_ phase/inhibited PMA-induced HCC invasion and apoptosis in human leukaemia cells HL-60 [34,35,36]
Camelledionol	Prenol lipids	Triterpenoids	41.48	441.34	440.33	C_29_ H_44_ O_3_	Inhibits the A549, LLC, HL-60, and MCF-7 cancer cell lines [55]
**Phenolics**							
Pterostilbene glycinate	Stilbenes	-	18.38	336.12	313.13	C_18_ H_19_ N O_4_	Effective in treat ment of melanoma and has anticancer activity [56]
Esculetin	Coumarins and derivatives	Hydroxycoumarins	13.08	177.02	178.03	C_9_ H_6_ O_4_	Inhibits migration and invasion of laryngeal cancer [45]
N-feruloyltyramine	Cinnamic acids and derivatives	Hydroxycinnamic acids and derivatives	19.03	312.12	313.13	C_18_ H_19_ N O_4_	Significantly fights against the oxidative damage induced by H_2_O_2_ and inhibits HepG2 cells [57]
**Quinones**							
Embelin	Prenol lipids	Quinone and hydroquinone lipids	27.50	293.18	294.19	C_17_ H_26_ O_4_	Inhibits TNF-α and cancer cell metastasis [46,47,48]
1,4-naphtho quinone	Naphthalenes	Naphthoquinones	14.04	159.04	158.04	C_10_ H_6_ O_2_	Its derivatives demonstrate good anticancer, antioxidant, antimicrobial, and anti-inflammatory effects [58,59]
**Flavonoids**							
Hesperetin	Flavonoids	o-methylated flavonoids	20.359	301.07	302.08	C_16_ H_14_ O_6_	A drug used for lowering cholesterol and treating multiple cancers [60]
Quercetin 3-(2″-p-hydroxybenzoyl -4″-p-coumaryl rhamnoside)	Flavonoids	Flavones	43.83	715.17	714.16	C_37_ H_30_ O_15_	Quercetin suppresses cyclooxygenase-2 (COX-2) expression in human breast cancer cells [54]

* Class and subclass were assigned according to the Human Metabolome Database (HMDB) (https://hmdb.ca) (accessed on 9 May 2022).

Although a high callus induction frequency was reported in all previously mentioned studies, only green or greenish-yellow compact calluses were produced, suggesting that the auxins and cytokinins used were not suitable for friable callus induction and proliferation in *R. nasutus*. The presence of green and compact calluses could be explained by the high concentration of cytokinin, which possibly promotes shoot and chlorophyll formation [61]. In contrast, our study showed for the first time that using two types of auxin (2,4-D and NAA) could effectively induce a bright yellow callus with a soft texture, possibly due to the presence of 2,4-D, which has been proposed to inhibit chlorophyll synthesis [62]. Additionally, NAA and subculturing for several cycles, as shown in our study, might play an important role in promoting friable callus formation, making it an attractive technique for establishing suspension cultures [13,63]. Similar results in other plants were reported by Anjusha et al. [31], who showed yellow friable callus induction on a medium containing only auxin (1 mg/L 2,4-D) from *Gynochthodes umbellata*, a medicinal plant belonging to family Rubiaceae, and from *Asteracantha longifolia* Nees, a medicinal plant belonging to the Acanthaceae family, which produced a friable white callus in the presence of auxin alone (2 mg/L 2,4-D) [64].

This research was the first attempt to establish a suspension culture of *R. nasutus*. The friable callus induced on the CIM containing 1 mg/L NAA and 1 mg/L 2,4-D was selected due to its fast-growing and bright-yellow color characteristics. Then, it was subcultured every two weeks for 21 subculture cycles until it was ready to initiate a cell suspension culture. The growth pattern of the *R. nasutus* suspension culture presented here was typical of the plant cell culture [65]. Slow growth was observed at the beginning of the cycle, then increases in both FW and DW were observed, potentially due to cell division and an increase in the water content of cells [66]. The highest bioactive compound accumulation was observed on day 15 of the culture, which was in agreement with the data from other authors [18,65]. The population of SCCs shrank after day 18, suggesting that cell death predominated during this period [67]. Based on the results from previous experiments, it was confirmed that the selected cell line was suitable for establishing a suspension culture and further investigating its antioxidant and anticancer properties compared to those of the leaf.

The current study revealed a higher TPC in the leaf extract of *R. nasutus* than reported in other studies based on the same method of extraction and solvent [5,68,69,70]. Similarly, the TFC obtained in the present study was comparable to those reported by other investigators [68,69,70]. Therefore, the solvent and extraction system are appropriate for the extraction of phenolic acids and flavonoids from *R. nasutus*. However, the TPC and TFC values of the SCC extract shown in the current study were relatively low compared to those of the leaf extract. Due to the presence of undifferentiated cells in calluses, it is possible that calluses can produce and accumulate lower levels of bioactive compounds than leaves, which are composed of fully differentiated cells. Notably, the suspension culture system is an effective system in which the bioactive compound accumulation can be substantially enhanced using elicitors, as observed in hairy root cultures of *R. nasutus* and other plants [28,71]. Since there are no reports comparing leaf and SCC extracts from *R. nasutus*, our study provides initial information on the established suspension culture system.

Much attention has been given to the antioxidant as well as other therapeutic properties of many medicinal herbs, including *R. nasutus*. The high antioxidant capacity of the leaf extract could be attributable to the high TPC observed in the present study, since the majority of the phenolic compounds have the ability to scavenge free radicals in many plant species, including *R. nasutus* [69,72,73]. Although flavonoids were detected in smaller amounts in the extract, evidence of many flavonoids contributing to the antioxidant activity was also reported [69]. Interestingly, although a lower TPC and TFC were observed in the SCC extract, the SCC extract exhibited a strong antioxidant potential equivalent to that of the leaf extract, as demonstrated by high scavenging activities of greater than 80% in the DPPH and ABTS assays as well as the high FRAP value. Thus, it is possible that other groups of phytochemicals, including carotenoids, alkaloids, terpenoids or quinones, present in the SCC extract contributed to the high antioxidant capacity, as demonstrated in other studies [4,5,74]. It should be noted that higher ABTS values may be attributed to the ABTS•^+^ (radical) stability over a wide pH range, the high sensitivity test, and the fast radical–antioxidant reaction. Moreover, ABTS•^+^ is reactive toward most antioxidants, supporting the evaluation of a wide range of antioxidants, including hydrophilic and lipophilic compounds [75,76] The observed antioxidant capacity levels of the *R. nasutus* leaf extract were in good agreement with the results from other investigators [69,72,77]. In summary, the SCC extract exhibited a high antioxidant potential equivalent to that of the leaf extract, suggesting the possibility of using the SCC extract in further applications.

In addition to the antioxidant capacity of the plant extract that is of particular interest, the potential for the plant extract to serve as an alternative or combined treatment for cancer is also worth exploring. Various other studies investigated the use of *R. nasutus* leaf and root extracts for cancer treatment. For the root extract, the previous studies showed effective inhibition of tumor cell growth in several cancer cell lines, including human cervical carcinoma (HeLa), human epithelial carcinoma (KB), human epidermoid carcinoma (Hep-2), human promyelocytic leukemia (HL-60), human uterus carcinoma (SiHa), human breast cancer (MCF-7), human colorectal adenocarcinoma (C-32, HT-29, Caco-2), human lung carcinoma (LLC, NCI-H187, A-549), mouse leukemia cell (P-388), and mouse colon adenocarcinoma (Colon-26) [8,78,79,80]. Additionally, the antiproliferative activity and cytotoxicity against HeLa cells, human prostate carcinoma cells (PC-3), human bladder carcinoma cells (T24), and multidrug-resistant CEM/ADR5000 cells were observed when applying the leaf extract [78,81,82].

This study provides the first evidence of the anticancer potential of *R. nasutus* leaf and SCC extracts against the oral cancer cell lines ORL-48 (oral cavity-gingiva) and ORL-136 (oral cavity-tongue). Based on the cancer cell viability and morphology, the leaf and SCC extracts could inhibit cancer cell proliferation, although the cytotoxic inhibitory effect of the SCC extract was lower than that of the leaf extract. Additionally, the SCC extract, which can be produced continuously in the laboratory, will also be beneficial in bioactive compound production for the treatment of oral cancer and other types of human cancer.

Considering the metabolites identified in the leaf and SCC extracts, the results obtained from this study are consistent with other reports of the presence of terpenoid, phenolic, flavonoid, and quinone compounds in *R. nasutus* leaves, conferring significant free radical scavenging activity and cancer-inhibitory effects [41,49,50,56,58,59,83,84]. Similar metabolite profiles were observed between the leaf and SCC extracts, with the leaf extract potentially showing greater metabolite accumulation. This information is not surprising, since the leaves at the mature stage normally accumulate several plant metabolites. In contrast, an undifferentiated callus may accumulate fewer types and smaller amounts of metabolites, depending on the selected cell line. However, some compounds, including camelledionol, pterostilbene glycinate, n-feruloyltyramine, 1,4-naphthoquinone, and hesperetin, were observed only in the SCC extract. The differences in metabolite accumulation in natural plant parts and those in the SCC might be attributed to various internal and external factors. In particular, callus induction and proliferation are under genetic control, so these factors might trigger differential gene expression, leading to different enzymes being involved in the biosynthesis of different compounds. In addition, external factors, including major and minor elements, hormones present in the soil and culture media, as well as the growing conditions (light, humidity, and temperature), are possible influences affecting the differences in metabolite production between the plant and the SCC [30].

Austroinulin isolated from *S. rebaudiana* has anti-inflammatory effects on nitric oxide (NO) production [32]. In addition, the ethanolic leaf extract of *S. rebaudiana* possessed antioxidant potential for use as a natural antioxidant agent [85]. Some terpenoid extracts of *G. lucidum* contain lucidenic acids with high antioxidant activity [86,87], and the impact of lucidenic acids (A and N) on cell growth inhibits PMA-induced invasion in hepatocellular carcinoma [34]. Esculetins, a class of coumarins and derivatives, and embelin, a naturally occurring benzoquinone obtained from the *Embelia ribes* Burm plant, possess a wide range of medicinal properties, including antioxidant, anticancer, anti-inflammatory, antibacterial, antifungal, and antiviral properties [45,46,47,48]. Additionally, the free radical scavenging reactions and antioxidant activity of embelin have been studied. Other studies in *R. nasutus* and *Citrus lumia* have reported that quercetin 3-(2″-p-hydroxy benzoyl-4″-p-coumarylrhamnoside) has antioxidant and anticancer potential [5,54]. Other compounds from *R. nasutus* are worth exploring in more detail, such as lignans, which significantly inhibited neuraminidase activity, suggesting their antiviral potential [7].

## 4. Materials and Methods

### 4.1. Plant Material

*R. nasutus* plants were grown and samples were collected in Khon Kaen Province, Thailand. The plants were identified at the Department of Biology, Faculty of Science, Khon Kaen University. The voucher specimen was deposited in the herbarium of the Department of Biology, Faculty of Science, Khon Kaen University (herbarium specimen number: KKU25555).

### 4.2. Explant Preparation

To prepare sterile plants for subsequent callus induction experiments, shoot tips of *R. nasutus* were used as explants. The shoot tips were washed with water with a few drops of dishwashing liquid for 5 min; after this, they were washed thoroughly under running tap water for 15 min. Inside a laminar flow cabinet (ESCO, Singapore), the shoot tips of *R. nasutus* plants (2.5 cm in length) were washed thoroughly with dishwashing liquid and running tap water and then dipped in 95% and 70% (*v*/*v*) ethanol for 1 and 4 min, respectively. After being washed three times in sterile distilled water, the shoot tips (5 mm in length) were treated with 20% and 10% (*v*/*v*) sodium hypochlorite (polysorbate 20 added) for 5 and 10 min, respectively. After five rinses with sterile distilled water, the surface-sterilized shoot tips were cultured on MS solid medium [88] supplemented with 3% (*w*/*v*) sucrose and 0.8% (*w*/*v*) Phytagel^TM^ at pH 5.7. The cultures were kept at 25 ± 2 °C under a light intensity of 2000 lux for 16/8 h light/dark.

### 4.3. Callus Induction, Cell Line Selection and Proliferation

Leaf segments, with dimensions of approximately 0.8 cm by 0.8 cm, developed from sterilized 6-week-old shoots were used as explants for callus induction. The CIM contained MS salts and vitamins with 3% (*w*/*v*) sucrose and 0.2% (*w*/*v*) Phytagel^TM^ supplemented with different concentrations and combinations of PGRs, i.e., 2,4-D (0.5, 1.0, 1.5, and 2.0 mg/L) and NAA (1.0 and 2.0 mg/L). The cultures were kept at 25 ± 2 °C with a light intensity of 2000 lux under a 16 h light/8 h dark photoperiod regime. The callus induction frequency (%), color, and texture, as visually assessed, were evaluated after 3 weeks of culture. The callus induction experiment was performed following a factorial design for each combination. Five leaf explants were placed into a jar for each treatment as one replicate, with three replicates per treatment. All experiments were repeated three times independently. After five weeks, the rapidly growing callus cell line was selected and subcultured on fresh CIM every two weeks.

### 4.4. Establishment of Cell Suspension Cultures and Growth Measurements

The fast-growing friable callus cell line with a creamy color was selected to establish a cell suspension culture. A preliminary test for the TPC was also performed. The growth of the friable callus was measured by sacrificial sampling. Two grams (FW) of a two-week-old friable callus was cultured in 250-mL Erlenmeyer flasks containing 100 mL of liquid CIM supplemented with 1 mg/L 2,4-D and 1 mg/L NAA. The cell suspensions were maintained at 110 rpm on a rotary shaker at 25 ± 2 °C, 2000 lux, and 16/8 h light/dark. To determine the cell suspension growth profile, the samples were harvested at 3-day intervals for a period of 30 days. All determinations were carried out in triplicate. The callus was separated from the medium by vacuum filtration, and the FW and DW were measured. Bright-field microscopy (Olympus, Tokyo, Japan) was used to examine the callus morphology. Evans blue staining was used to observe the cell viability [67].

### 4.5. Preparation of Extracts

Leaf and SCC samples of *R. nasutus* were dried at 45 °C for 24 h and then ground into a fine powder. One gram of each sample powder was subjected to maceration extraction using 50 mL of 80% (*v*/*v*) ethanol at 65 °C for 6 h. The supernatant was collected and then filtered through filter paper. The solvent was evaporated (EYELA N-1100, Tokyo, Japan) and further lyophilized (Christ, Osterode, Germany). The obtained dried powder was stored at −20 °C until further use in subsequent experiments. For the analysis of plant metabolites by UHPLC–QToF–MS), dried samples (15% *w*/*v*) were used following the maceration extraction procedure previously mentioned.

### 4.6. Determination of the TPC and TFC

The TPC values of leaf and SCC extracts were estimated using the FC method, following the method described by Folin and Ciocalteu [89] with slight modifications. Briefly, twenty microliters (20 μL) of each extract (10 mg/mL) was mixed with 60 μL of distilled water and 20 μL of FC reagent (1 N). After 5 min, 100 μL of 8% (*w*/*v*) sodium carbonate (Na_2_CO_3_) was added and the final volume was adjusted to 300 μL using distilled water. The reaction mixture was incubated for 30 min at 25 °C in the dark. The absorbance at 765 nm was measured using a microplate reader (Thermo Scientific Varioskan^®^ Flash, Waltham, MA, USA). Gallic acid was used as a reference, and the results are presented as milligrams of gallic acid equivalents per gram of dry weight (mg GAE/g DW).

For TFC determination, the aluminum chloride (AlCl_3_) colorimetric assay as described by Zongo et al. with slight modifications was employed [90]. In brief, 100 μL each of the leaf and SCC extracts (2 mg/mL) was mixed with 100 μL of 2% AlCl_3_. The mixed reactions were incubated for 1 h at 25 °C in the dark before the absorbance of the mixture was assayed at 420 nm. Quercetin was chosen as the standard, and the TFC was expressed as quercetin equivalents in milligrams per gram of dry sample (mg QE/g DW).

### 4.7. Determination of Antioxidant Capacity

Three methods, namely the FRAP assay (OxiSelect™ Ferric-Reducing Antioxidant Power Assay Kit, Cell Biolabs, San Diego, CA, USA), ABTS assay (OxiSelect™ Trolox Equivalent Antioxidant Capacity Assay Kit, Cell Biolabs) and DPPH assay (Sigma, Ronkonkoma, NY, USA), were used to evaluate the total antioxidant capacity of leaf and SCC extracts. The experiments were carried out using 96-well plates, and the absorbance at specific wavelengths was measured using a microplate reader (Thermo Scientific Varioskan^®^ Flash, Waltham, MA, USA) according to the methodology described by Benzie et al. (1996), Smeriglio et al. (2017), and Shalaby and Shanab (2013) with some modifications.

The method used by Benzie et al. [91] was adapted to determine the antioxidant capacity using the FRAP assay with slight modifications. Briefly, 100 μL of each extract (2 mg/mL) was mixed with 100 μL of FRAP reagent and incubated for 10 min at 25 °C, then the absorbance was read at 540 nm. Iron (II) oxide (Fe^2+^) served as the standard (0–500 μM), and the FRAP values are expressed as μM Fe^2+^/g DW.

For the ABTS assay with Trolox as the reference compound, the ABTS•+ radical solution was generated by mixing 4.3 mM (*w*/*v* in water) potassium persulfate (K_2_S_2_O_8_) and 1.8 mM ABTS solution at a ratio of 1:5 (*v*/*v*). The mixtures were incubated for 1 h at 25 °C in the dark. Before reacting with the samples, the ABTS•+ radical solution was diluted with distilled water to obtain an absorbance of 0.7 ± 0.02 at 734 nm. Then, ten microliters of each extract (2 mg/mL) was added to 200 μL of ABTS•+ solution, the mixture was incubated in the dark for 6 min, and the absorbance at 734 nm was recorded [92].

The DPPH assay was performed according to Shalaby and Shanab [93] with some modifications. Ten milligrams of leaf and SCC extracts was dissolved in 1 mL of distilled water. Then, 100 µL of each sample was reacted with 100 µL of DPPH radical (0.1 mM in methanol). The mixtures were shaken vigorously and then kept for 30 min at 25 °C. All determinations were conducted with three replicates using Trolox as the standard equivalent (0 to 500 mg/L). The absorbance at 515 nm was measured, and the antioxidant capacity of the extracts was calculated as follows.

The antioxidant capacity of the extracts was calculated using ABTS and DPPH assays. The percent inhibition of radicals was calculated as the % scavenging activity using the following equation:

% Scavenging activity = [(A control − A sample) × 100]/A control

where A control is the absorbance of the ABTS or DPPH radical and A sample is the absorbance of the ABTS or DPPH radical and sample.

### 4.8. Determination of Anticancer Activity

The effects of leaf and SCC extracts on oral cancer cell viability were determined using the 3-(4,5-dimethylthiazol-2-yl)-2,5-diphenyltetrazolium bromide (MTT) assay. Briefly, ORL-48 and ORL-136 cell lines [94] were seeded into a 96-well culture plate at a density of 1.9 × 10^4^ cells/well and cultured in Dulbecco’s modified Eagle’s medium–Nutrient Mixture F-12 (DMEM/F12) containing 10% fetal bovine serum, 0.4 µg/mL of hydrocortisone, and 1% antibiotic-antimycotic. After 24 h of incubation at 37 °C in a fully humidified atmosphere of 5% CO_2_, the oral cancer cell lines were treated with various concentrations of leaf and SCC extracts (1, 2, 4, 6, and 8 mg/mL) for 48 h. Then, the MTT solution (5 mg/mL MTT in phosphate-buffered saline, PBS) was added and the samples were incubated for 3.5 h. Next, all solutions were removed. The formazan product in each well was solubilized with dimethyl sulfoxide (DMSO) and the plate was incubated for 10 min. The absorbance was measured using a microplate reader at 570 nm. The percentage of cancer cell viability was determined based on a comparison with untreated cells grown in a complete medium.

The IC_50_ value, the concentration of the test extract required for 50% cell viability, was determined from the plot between the % cell viability and extract concentration. The average IC_50_ value was then calculated.

### 4.9. UHPLC–QToF–MS

#### 4.9.1. Data Acquisition

The dried leaf and SCC extracts were reconstituted in isopropanol and were analyzed using a UHPLC system coupled with QToF–MS (Agilent, Santa Clara, CA, USA). Briefly, the samples were injected into a UHPLC system equipped with a Zorbax Eclipse Plus C18 column (150 × 2.1 mm, 1.8 µm, Agilent) and with the diode array detector (DAD). The column temperature was set to 25 °C. The elution gradient was implemented with a binary solvent system consisting of water (LC–MS grade) with 0.1% formic acid (solvent A) and acetonitrile (LC–MS grade) with 0.1% formic acid (solvent B). The elution gradient was set as follows: 100% A (0–2 min); 0% A (45–50 min) with a flow rate of 0.2 mL/min. Peaks were detected at wavelengths of 254, 280, and 360 nm. The MS spectra were acquired in both positive and negative electrospray ionization (ESI) modes by employing two and five microliters of each sample injection, respectively. The MS system was set to 325 °C, with desolvation flow gas at 13 L/min and TOF MS with electrospray ionization as the ion source. The ionization voltages in positive and negative polarity modes were 2000 and 2000 V, respectively. Data were acquired in the profile mode for 50 min, and the mass ranges were set to 100–1200 m/z for MS and 50–1200 m/z for MS/MS.

#### 4.9.2. Data Processing

All spectra were processed using MassHunter workstation software qualitative analysis workflows V8 and MassHunter workstation software qualitative analysis navigator V8. Public databases, namely Metlin (https://metlin.scripps.edu, accessed on 12 May 2022), Human Metabolome Database (HMDB) (https://hmdb.ca) (accessed on 12 May 2022), and Kyoto Encyclopedia of Genes and Genomes (KEGG) (https://www.genome.jp/kegg/) (accessed on 12 May 2022), were also used.

### 4.10. Statistical Analysis

All experimental data were subjected to ANOVA followed by Duncan’s multiple range test (DMRT) and the independent-samples *t* test using SPSS software version 19 with a *p* value of 0.05 (*p* ≤ 0.05). The results were expressed as the means ± standard deviations (SDs) of three measurements. Graphing, calculations, and statistical analyses were performed using GraphPad Prism 9 software version 9.3.1 (GraphPad Software, San Diego, CA, USA). The correlations among the data obtained were calculated using MS Excel software through the correlation coefficient statistical function.

## 5. Conclusions

This is the first report on establishing callus and cell suspension cultures of *R. nasutus* using in-vitro-derived leaf explants. Two PGRs, 2,4-D and NAA, were found to be essential for yellow friable callus induction and the proliferation of *R. nasutus*. Cultures obtained on day 15 are suggested for harvesting in order to obtain healthy and viable cells for bioactive compound production. The leaf extract exhibited higher FRAP and DPPH values than the SCC extract, whereas the ABTS values showed comparable antioxidant capacities between the leaf and SCC extracts. Although the SCC extract exhibited slightly lower anticancer activity than the leaf extract, it had a potential inhibitory effect against the oral cancer cell lines ORL-48 (oral cavity-gingiva) and ORL-136 (oral cavity-tongue). The UHPLC–QToF–MS analysis confirmed that the detected secondary metabolites in the leaf and SCC extracts were terpenoids, phenolics, quinones, and flavonoids. Further investigations using elicitors to induce bioactive compound production and optimizing several parameters for phytochemical production using cell suspension culture should be explored. Detailed metabolite profiling and additional identification of the leaf and SCC extracts after optimization are required to detect the presence of other compounds that might play an essential role in the antioxidant and anticancer potential of *R. nasutus*. Moreover, an assessment of the anticancer effect using other cell viability assays, apoptosis assays, cell migration, and cell cycle progression by flow cytometry should be considered.

## Figures and Tables

**Table 1:** The effects of different combinations of plant growth regulators on callus induction in *R. nasutus*.

2,4-D (mg/L)	NAA (mg/L)	% Callus Induction *	Callus Response
0	0	0 ^c^	no growth response
0.50	1.00	100.00 ± 0.00 ^a^	friable, yellow
1.00	1.00	100.00 ± 0.00 ^a^	friable, yellow
1.50	1.00	100.00 ± 0.00 ^a^	friable, yellow
2.00	1.00	100.00 ± 0.00 ^a^	friable, yellow
0.00	1.00	26.67 ± 0.58 ^b^	compact, yellow
0.00	2.00	0 ^c^	no growth response
0.50	2.00	33.33 ± 0.58 ^b^	compact, yellow
1.00	2.00	33.33 ± 0.58 ^b^	compact, yellow
1.50	2.00	26.67 ± 0.58 ^b^	compact, yellow
2.00	2.00	26.67 ± 0.58 ^b^	compact, yellow
1.00	0.00	0 ^c^	no growth response
2.00	0.00	0 ^c^	no growth response

* The data are presented as the means ± standard deviation (SD) from three independent experiments. Mean values in the column followed by different superscripts (a, b or c) are significantly different according to the analysis of variance (ANOVA) with Duncan’s multiple range test (DMRT) at the level of 0.05 (*p* ≤ 0.05).

## Data Availability

The datasets analyzed during the current study are available from the corresponding author upon reasonable request.

## Supplementary Materials

The following supporting information can be downloaded at: https://www.mdpi.com/article/10.3390/plants11151994/s1. Figure S1: Total phenolic content of *R. nasutus* cell suspension cultures on days 9, 15, and 18. Figure S2: Morphology of ORL-48 and ORL-136 cell lines treated with the leaf extract. Figure S3: Morphology of ORL-48 and ORL-136 cell lines treated with the SCC extract. Figure S4: MS spectra of compounds detected in leaf and SCC extracts. Table S1: List of compounds identified in *R. nasutus* leaf extract by UHPLC–QToF–MS analysis. Table S2: List of compounds identified in *R. nasutus* callus extract by UHPLC–QToF–MS analysis. Table S3: Class and subclass of metabolites found in *R. nasutus* leaf and SCC extracts by UHPLC–QToF–MS analysis.
