# Supplementary material for: Antioxidant and Anticancer Potential of Bioactive Compounds from Rhinacanthus nasutus Cell Suspension Culture
Source: Plants (Basel). 2022 Jul 30;11(15):1994. doi: 10.3390/plants11151994 (PMC9370634; doi:10.3390/plants11151994)
Supplement: Supplementary file 1 [file plants-11-01994-s001.zip › plants-1811370-supplementary.pdf]

Supplementary Figures

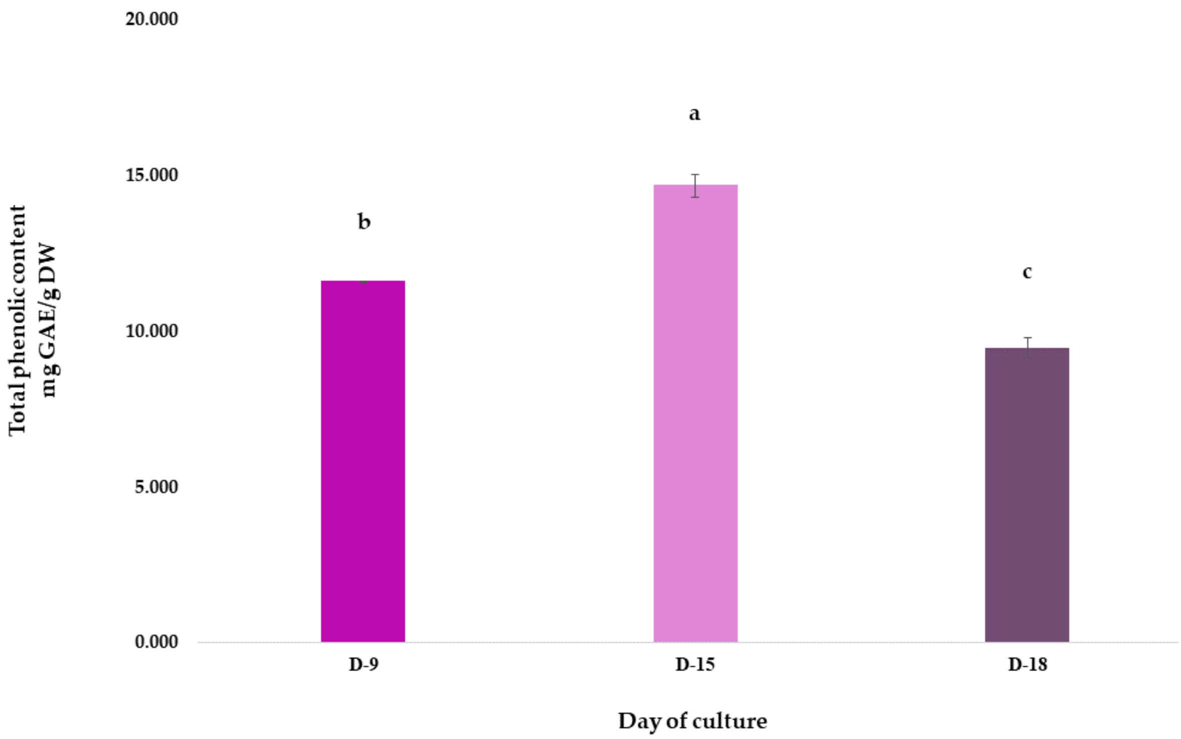

Figure S1. Total phenolic content of *R. nasutus* cell suspension cultures on day 9, 15 and 18. The data are the results performed in triplicate. Each value followed by the different superscripts are significantly different using post hoc test and ANOVA with Duncan's multiple range test (DMRT) at the level of 0.05 ($p \leq 0.05$).

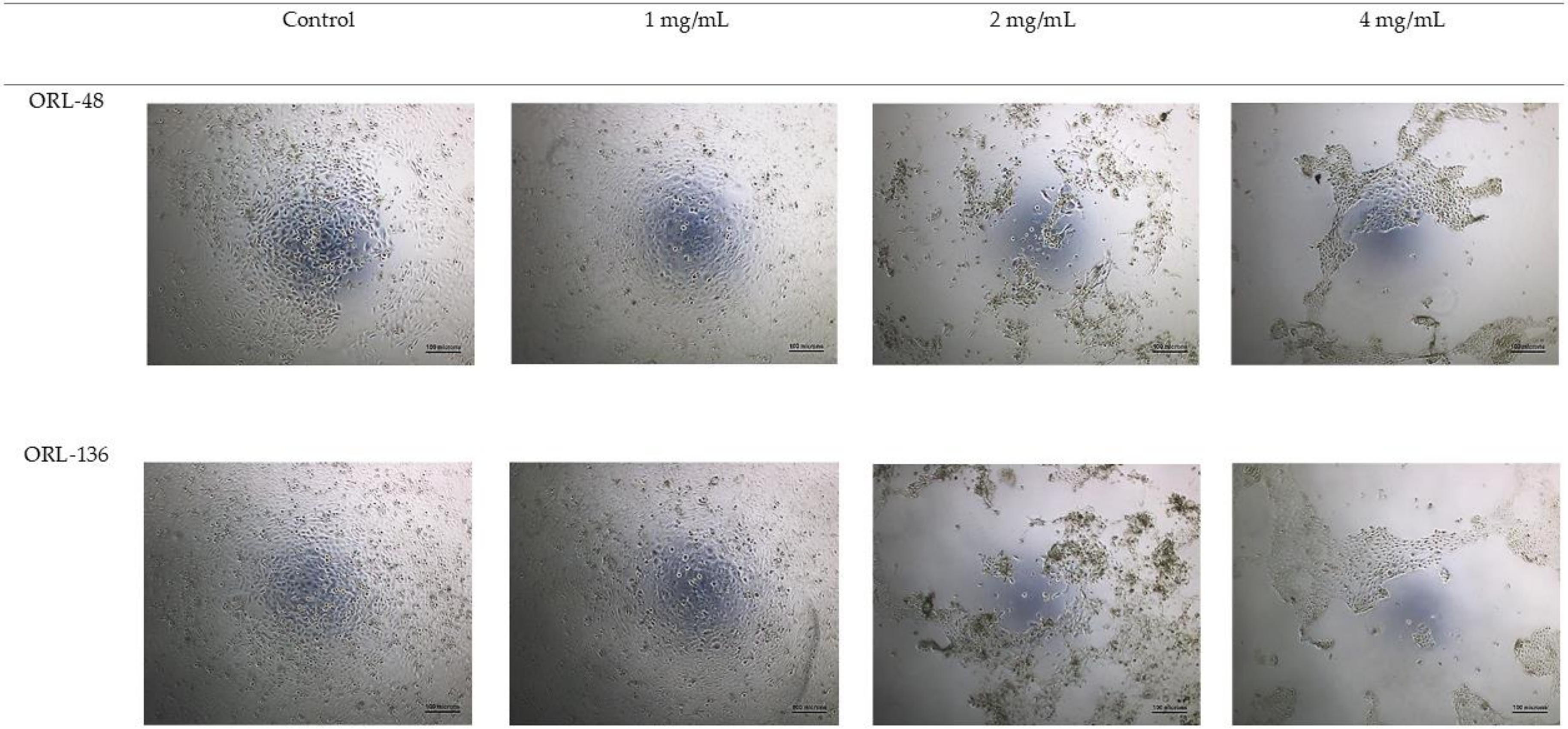

Figure S2. Morphology of ORL-48 and ORL-136 cell lines treated with the leaf extract. The scale bar represents 100 μm.

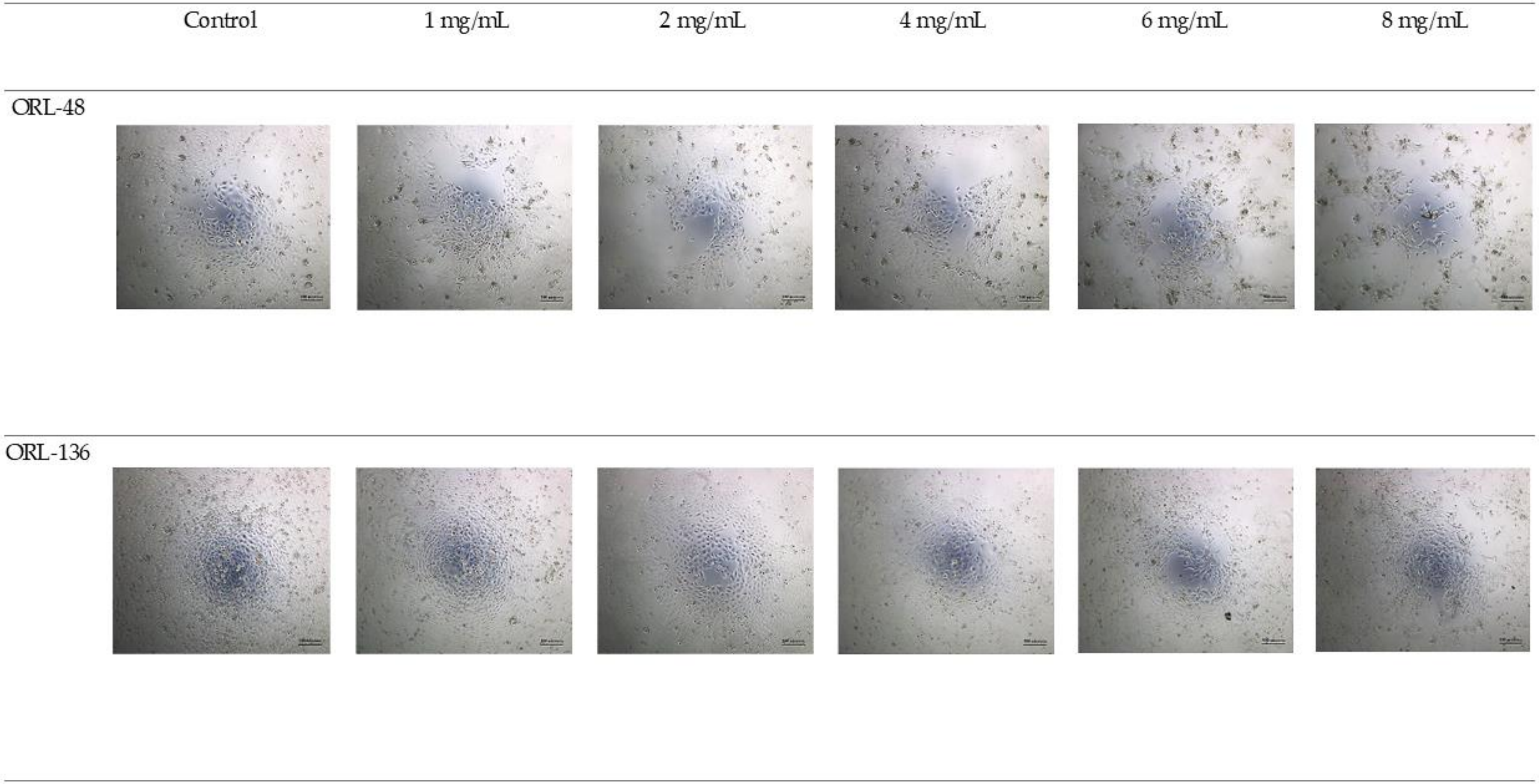

Figure S3. Morphology of ORL-48 and ORL-136 cell lines treated with the SCC extract. The scale bar represents 100 μm.

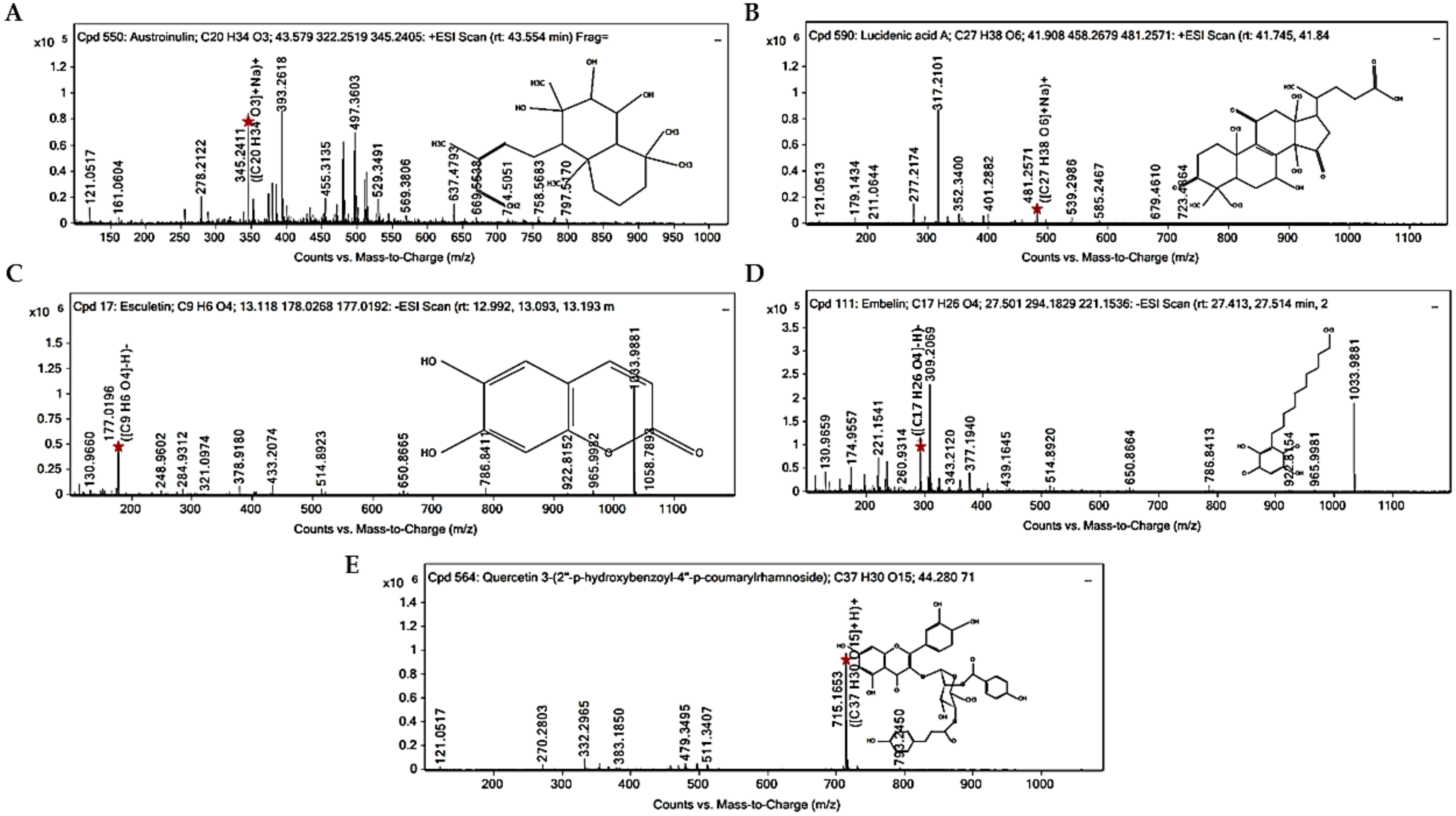

Figure S4. MS spectra of compounds detected in leaf and SCC extracts. (A) Austroinulin, (B) lucidenic acid, (C) esculetin, (D) embelin and (E) quercetin 3-(2''-p-hydroxybenzoyl-4''-p-coumarylrhamnoside. A star represents the detected compounds.

Supplementary Tables

Table S1. List of compounds identified in *R. nasutus* leaf extract by UHPLC-QToF-MS analysis

Table S1A. Hexane partition [positive mode]

Compound	Class	Subclass	Molecular Formula	Retention Time (min)	Mass	Product Ions (m/z)
(R)-Bitalin A	19	19.1	C13 H14 O3	16.543	218.0936	219.1009
His Glu Lys	4	-	C17 H28 N6 O6	21.429	412.2061	413.2134
Ser Arg Pro	4	-	C14 H26 N6 O5	21.579	358.1974	359.2047
Acetyl Tyrosine Ethyl Ester	-	-	C13 H17 N O4	23.96	251.1166	252.1239
(4E,8E,10E-d18:3)sphingosine	-	-	C18 H33 N O2	24.16	295.2501	296.2574
Palmitic amide	1	1.1	C16 H33 N O	24.185	255.2572	256.2645
C16 Sphinganine	5	5.1	C16 H35 N O2	24.749	273.2677	274.275
3-Ethyltridecan-2-one	-	-	C15 H30 O	24.937	226.2306	244.2644
Hexadecan-3-one	-	-	C16 H32 O	25.764	240.2464	258.2803
U-46619 Glycine methyl ester	-	-	C24 H39 N O5	26.04	421.2835	422.2907
Sodium glycocholate	3	3.1	C26 H43 N O6	26.24	465.3105	466.3179
Granisetron	19	19.2	C18 H24 N4 O	26.465	312.1944	335.1837
2',4'-Dihydroxy-7-methoxy-8-prenylflavan	17	17.1	C21 H24 O4	26.691	340.167	363.1562
N-acetyltryptophan	6	6.1	C13 H14 N2 O3	26.741	246.1012	247.1085
Gingerenone B	19	19.3	C22 H26 O6	26.892	386.174	409.1633
Methyl nigakinone	19	-	C16 H12 N2 O3	27.242	280.0857	281.093
Safficinolide	19	-	C20 H24 O5	27.343	344.1623	345.1694
Phytosphingosine	5	5.1	C18 H39 N O3	27.468	317.294	318.3012
beta-Zearalanol	16	-	C18 H26 O5	27.643	322.1792	345.1683
Spisulosine	5	5.1	C18 H39 N O	27.668	285.3041	286.3114
Maculosin	6	6.1	C14 H16 N2 O3	27.844	260.1173	261.1246
Rosoxacin	14	14.1	C17 H14 N2 O3	28.044	294.1016	295.1089
Lys Ser Asp	4	-	C13 H24 N4 O7	28.695	348.1641	349.1713
Auxin b	2	2.1	C18 H30 O4	28.946	310.2157	333.205
PGF2α diethyl amide	-	-	C24 H43 N O4	28.996	409.3203	410.3275
15-methyl-15(S)-PGE1	-	-	C21 H36 O5	29.397	368.2569	391.2467
Prosopinine	19	-	C16 H33 N O3	29.497	287.2471	310.2364
4,6'-Epoxyorotiniflavan-4-ol	-	-	C26 H28 O6	29.598	436.1882	437.1957
Chamuvaritin	-	-	C29 H24 O5	29.635	452.1615	453.1692
(ent-16betaOH)-16,17-Dihydroxy-9(11)-kauren-19-oic acid	7	-	C20 H30 O4	30.049	334.2136	335.2208
Imazamethabenz	6	6.1	C15 H18 N2 O3	30.374	274.133	275.1403
Alfuzosin	18	18.1	C19 H27 N5 O4	30.474	389.2073	407.2412
Cincassiol B	2	2.2	C20 H32 O8	30.55	400.2108	423.2001
(S)-Verimol F	19	19.4	C17 H20 O3	30.75	272.1422	295.1312
N,N-dimethyl-Safingol	-	-	C20 H43 N O2	30.875	329.3302	330.3375
3,3',4,4'-Tetrahydroxy-5,5'-diisopropyl-2,2'-dimethylbiphenyl	19	19.5	C20 H26 O4	31.151	330.1831	331.1901
Dihydroxycarteolol M2	-	-	C16 H24 N2 O5	31.201	324.1681	347.1573
Leu Ala Arg	4	-	C15 H30 N6 O4	31.301	358.2346	359.2419
Methyl cis-p-coumarate 3-(3,7-dimethyl-2,6-octadienyl)	12	12.1	C20 H26 O3	31.439	314.1874	315.1945
Glabrone	19	19.6	C20 H16 O5	31.677	336.0989	337.1061
Stearidonic Acid	1	1.2	C18 H28 O2	31.878	276.2102	277.2175
Ser Val Gln	4	-	C13 H24 N4 O6	32.479	332.1683	333.1754
ketophenylbutazone	16	-	C19 H18 N2 O3	32.629	322.1326	323.1397
(±)-Rollipyrrole	16	-	C16 H20 N2 O3	32.767	288.1483	289.1557

Compound	Class	Subclass	Molecular Formula	Retention Time (min)	Mass	Product Ions (m/z)
Citronellyl alpha-toluate	1	1.3	C18 H26 O2	33.018	274.1946	275.2019
Pro Ser Gln	4	-	C13 H22 N4 O6	33.782	330.1526	331.1598
12-Hydroxy-7-oxo-8,11,13-abietatrien-18-al	2	2.2	C20 H26 O3	33.782	314.188	315.1952
Acetylenic acids; 17-Octadecen-9-ynoic acid	1	1.4	C18 H30 O2	34.007	278.225	279.2322
Gln Val Ala	4	-	C13 H24 N4 O5	34.358	316.1735	317.1806
11β-Prostaglandin F1β	1	1.5	C20 H36 O5	34.458	356.2556	357.263
Lisuride	14	14.2	C20 H26 N4 O	34.508	338.2101	361.2001
8-HpODE	1	1.2	C18 H32 O4	34.559	312.2293	313.2367
Isocordoin	-	-	C20 H20 O3	34.621	308.14	309.1472
Nb-Feruloyltryptamine	12	12.2	C20 H20 N2 O3	34.784	336.1484	337.1556
9-Docosene	19	19.7	C22 H44	34.872	308.3458	326.3797
trans,trans-Farnesyl phosphate	-	-	C15 H27 O4 P	35.16	302.1648	303.1722
ent-8-deoxy-J2-IsoP	-	-	C20 H28 O3	35.485	316.2024	317.2103
α-Linolenoyl Ethanolamide	5	5.1	C20 H35 N O2	35.661	321.2678	344.257
Tris(butoxyethyl)phosphate	19	19.8	C18 H39 O7 P	35.862	398.2443	421.2337
Scorzoside	2	2.3	C21 H30 O8	35.949	410.1946	433.1838
17-phenyl-trinor-PGE2	1	1.5	C23 H30 O5	36.162	386.2107	409.1999
1,2,3,4-Tetrahydro-1-[1-hydroxy-3-(4-hydroxyphenyl)-2-propenyl]-7-methoxy-2,6-naphthalenediol	19	-	C20 H22 O5	36.287	342.1473	365.1366
11-deoxy-PGE1	1	1.5	C20 H34 O4	36.751	338.2472	361.2361
Gancaonin V	15	15.1	C19 H20 O4	36.913	312.1371	335.1262
Epanolol	11	-	C20 H23 N3 O4	37.214	369.1692	392.1584
Ergine	-	-	C16 H17 N3 O	37.315	267.1367	285.1711
Cyclopassifloside II	2	2.4	C37 H62 O11	37.515	682.4265	683.434
Verimol C	11	11.1	C18 H20 O4	37.741	300.1357	301.1429
Crucigasterin 277	-	-	C18 H31 N O	37.816	277.2415	278.2491
9-hydroxy-13-oxo-10-octadecenoic acid	1	1.2	C18 H32 O4	37.841	312.2313	335.2205
PA(22:6(4Z,7Z,10Z,13Z,16Z,19Z)/17:1(9Z))	19	19.9	C42 H69 O8 P	38.292	732.474	367.2455
Androst-4-ene-3alpha,17beta-diol diacetate	-	-	C23 H34 O4	38.304	374.2446	375.2519
(25S)-5alpha-cholestan-3beta,4beta,6alpha,8beta,15alpha,16beta,26-heptol	-	-	C27 H48 O7	38.442	484.34	507.3297
Leu Trp Lys	4	-	C23 H35 N5 O4	38.517	445.2695	463.3033
Simvastatin acid	19	19.10	C25 H38 O5	38.693	418.2704	419.2778
Montanol	1	1.4	C21 H36 O4	38.818	352.2624	375.2516
Deoxycorticosterone	3	3.2	C21 H30 O3	38.893	330.218	331.2253
Piperochromenoic acid	2	2.1	C22 H28 O3	39.332	340.2048	363.1942
Polidocanol	8	8.1	C30 H62 O10	39.444	582.4348	605.4241
Digitoxigenin	3	3.3	C23 H34 O4	39.444	374.2445	375.2518
Ser Ser Arg	4	-	C12 H24 N6 O6	39.632	348.1751	371.1645
17,20-dimethyl Prostaglandin F1α	1	1.5	C22 H40 O5	39.707	384.2857	385.293
13-methoxy-heneicosanoic acid	-	-	C22 H44 O3	39.795	356.3294	379.3187
N-cis-octadec-9Z-enoyl-L-Homoserine lactone	-	-	C22 H39 N O3	39.92	365.2924	366.2996
Piperoic acid	2	2.5	C22 H30 O4	40.02	358.2138	359.2211
14,15-HxA3 (11S)	-	-	C20 H32 O4	40.196	336.2297	337.237

Compound	Class	Subclass	Molecular Formula	Retention Time (min)	Mass	Product Ions (m/z)
16-Hydroxy-10-oxohexadecanoic acid	1	1.4	C16 H30 O4	40.321	286.215	309.2043
Medroxyprogesterone	19	19.11	C22 H32 O3	40.397	344.234	345.2411
Thromboxanoic acid skeleton	-	-	C20 H36 O4	40.635	340.2615	341.2689
8beta-Angeloyloxy-15-hydroxy-1alpha,10R-dimethoxy-3-oxo-11(13)-germacren-12,6alpha-olide	2	2.3	C22 H32 O8	40.747	424.2094	425.2166
12(13)-EpOME	1	1.4	C18 H32 O3	40.747	296.2347	297.2421
Austalide I	9	9.1	C27 H34 O8	40.873	486.2252	487.2329
20,24-Epoxy-25,26-dihydroxydammaran-3-one	2	2.4	C30 H50 O4	40.998	474.3716	497.3607
Capnine	6	6.1	C17 H37 N O4 S	41.123	351.2444	369.2781
Quasiprotopanaxatriol	2	2.6	C30 H50 O3	41.223	458.3759	481.3654
PI(13:0/0:0)	-	-	C22 H43 O12 P	41.323	530.2506	531.2581
Asclepin	1	1.4	C31 H42 O10	41.511	574.2775	575.2849
Cortexolone	3	3.2	C21 H30 O4	41.9	346.2142	347.2216
Acetyl-11-keto-B-Boswellic Acid, 3-	2	2.6	C32 H48 O5	41.912	512.3482	513.3556
Schleicherastatin 5	3	3.4	C29 H48 O3	42.201	444.361	445.3683
2E-Phytenoic acid	-	-	C20 H38 O2	42.426	310.2887	311.2959
1-Oleoyl-2-acetyl-sn-glycerol	-	-	C23 H42 O5	42.476	398.3024	399.3098
AT-56	-	-	C25 H27 N5	42.627	397.2273	420.2166
Methyl acetyl ricinoleate	-	-	C21 H38 O4	42.726	354.2759	355.2832
N-Desalkylbuprenorphine	15	-	C25 H35 N O4	42.777	413.2552	414.2626
PI(15:0/16:0)	-	-	C40 H77 O13 P	42.852	796.5098	797.5173
(ent-2b,4S,9a)-2,4,9-Trihydroxy-10(14)-oplopen-3-one 2-(2-methylbutanoate) 9-(3-methyl-2E-pentenoate)	2	2.5	C26 H40 O6	43.228	448.2822	449.2895
Corosolic acid	2	2.6	C30 H48 O4	43.303	472.3561	495.3453
3-Epimasticadienolic acid	2	2.6	C30 H48 O3	43.378	456.3606	479.3498
Austroinulin	2	2.2	C20 H34 O3	43.579	322.2519	345.2411
Auricularine	-	-	C33 H42 N4	43.591	494.3429	495.3462
1-Naphthylacetylspermine	-	-	C22 H34 N4 O	43.604	370.2726	393.2618
Ganodermic acid TQ	16	-	C32 H46 O5	43.704	510.3365	511.3406
3α,6β,12α-Trihydroxy-5β-cholan-24-oic Acid	-	-	C24 H40 O5	43.829	408.2856	409.293
Harderoporphyrin	10	10.1	C35 H36 N4 O6	44.08	608.2648	609.2722
9alpha-(3-Methyl-2E-pentenoyloxy)-4S-hydroxy-10(14)-oplopen-3-one	2	2.5	C21 H32 O4	44.18	348.2299	349.2372
Quercetin 3-(2''-p-hydroxybenzoyl-4''-p-coumarylrhamnoside)	17	17.5	C37 H30 O15	44.28	714.1578	715.1653
N,N-dimethyl arachidonoyl amine	-	-	C22 H37 N O	44.343	331.2891	332.2964
Atocalcitol	-	-	C32 H46 O4	44.443	494.3412	495.3452
11beta-Hydroxy-5beta-cholan-24-oic Acid	-	-	C24 H40 O3	44.631	376.2975	377.3049
Resolvin D2	1	1.4	C22 H32 O5	44.982	376.2237	399.2137
Paratocarpin E	-	-	C25 H28 O5	45.032	408.1949	431.1848
7-Ketodeoxycholic acid	3	3.1	C24 H38 O5	45.132	406.2713	407.2786
(13R,14R)-7-Labdene-13,14,15-triol	2	2.2	C20 H36 O3	45.508	324.2657	325.2731

Compound	Class	Subclass	Molecular Formula	Retention Time (min)	Mass	Product Ions (m/z)
(24E)-3alpha-Acetoxy-15alpha,22S-dihydroxylanosta-7,9(11),24-trien-26-oic acid	2	2.6	C32 H48 O6	45.658	528.3442	529.3515
Pheophorbide a	-	-	C35 H36 N4 O5	45.696	592.2707	593.278
Santalyl phenylacetate	2	2.5	C23 H30 O2	45.784	338.2255	356.2593
Polyporusterone C	3	3.5	C28 H44 O6	46.034	476.315	477.3219
3alpha,7alpha,12alpha-Trihydroxy-5beta-23E-cholestan-26-oic acid	1	1.6	C26 H42 O5	46.134	434.303	435.3106
3β-Acetoxydeoxyangolensic Acid, Methyl Ester	-	-	C29 H38 O8	46.147	514.2572	515.2649
PI(15:0/0:0)	-	-	C24 H47 O12 P	46.209	558.2823	559.29
Tigecycline	13	-	C29 H39 N5 O8	46.41	585.2819	603.316
Palmitoyl N-Isopropylamide	-	-	C19 H39 N O	46.71	297.3038	298.3111
DG(22:5(7Z,10Z,13Z,16Z,19Z)/14:0/0:0)	19	19.12	C39 H66 O5	46.936	614.4893	637.479
Lepidiumterpenyl ester	2	2.5	C23 H42 O4	47.036	382.3078	383.315
2-Deoxybrassinolide	3	3.3	C28 H48 O5	47.087	464.3491	465.3564
PGF2α Alcohol	-	-	C20 H36 O4	47.236	340.2624	363.2516
Polyporusterone F	3	3.1	C28 H46 O5	47.337	462.335	463.3423
Persenone B	1	1.7	C23 H42 O4	47.437	382.3079	383.3156
N-Hexadecanoylpyrrolidine	19	19.13	C20 H39 N O	47.512	309.3039	310.3112
4,4'-Diapophytofluene	-	-	C30 H46	47.612	406.3605	407.3676
Coenzyme Q4	2	2.7	C29 H42 O4	47.637	454.3074	455.3146
27-Nor-5b-cholestane-3a,7a,12a,24,25-pentol	3	3.1	C26 H46 O5	47.713	438.3355	461.3242
Vitamin D3 glucosiduronate	3	3.6	C33 H52 O7	48.013	560.3698	561.3772
3alpha,7alpha,12alpha,24-tetrahydroxy-24-methyl-5beta-cholestan-26-oic acid	1	1.6	C28 H48 O6	48.064	480.346	481.3534
2-(9R-(tricosanoyloxy)-3-methyl-2Z-decenoyloxy)-ethanesulfonic acid	-	-	C36 H68 O7 S	48.164	644.4674	667.4566
Haplophytine	-	-	C37 H40 N4 O7	48.239	652.2913	653.2986
Hexacosanedioic acid	-	-	C26 H50 O4	48.314	426.3707	449.36
Harderoporphyrinogen	10	10.1	C35 H42 N4 O6	48.515	614.3132	637.3026
Momordol	1	1.7	C26 H48 O5	48.74	440.3483	441.3556
Theasapogenol A	2	2.6	C30 H50 O6	48.84	506.3589	507.3662
28-Homobrassinolide	3	3.3	C29 H50 O6	48.865	494.3617	517.3511
6-Deoxodolichosterone	3	3.1	C28 H48 O4	48.991	448.3548	449.362
12β-Hydroxy-3-oxo-5β-cholan-24-oic Acid	3	3.1	C24 H38 O4	49.066	390.2782	413.2675

Table S1B. Hexane partition [negative mode]

Compound	Class	Subclass	Molecular Formula	Retention Time(min)	Mass	Product Ions (m/z)
3-Hydroxycoumarin	24	24.1	C9 H6 O3	15.937	162.0316	161.0243
Kamahine C	8	8.1	C14 H20 O5	21.975	268.1312	267.124
2,2,4,4-Tetramethyl-6-(1-oxobutyl)-1,3,5-cyclohexanetrione	2	2.1	C14 H20 O4	24.155	252.1362	251.129
Methylhalfordinol	19	19.15	C15 H12 N2 O2	26.122	252.0903	311.1039
Embelin	2	2.7	C17 H26 O4	27.413	294.1835	293.1763
SU 5416	-	-	C15 H14 N2 O	28.039	238.1108	297.1247
9-hydroperoxy-10E,12,15Z-octadecatrienoic acid	1	1.2	C18 H30 O4	28.265	310.2144	309.207
(±)9-HpODE	1	1.2	C18 H32 O4	30.194	312.2301	311.2228
5-O-Methylembelin	2	2.7	C18 H28 O4	31.296	308.1988	307.1913
13(S)-HOTrE	1	1.2	C18 H30 O3	31.948	294.2199	293.2127
9(S)-HOTrE	1	1.2	C18 H30 O3	32.249	294.2202	293.2131
2',4',6',3,4-Pentahydroxy- 3',5-diprenyldihydrochalcone	19	19.16	C25 H30 O6	32.499	426.204	425.1966
alpha-licanic acid	-	-	C18 H28 O3	33.05	292.2044	291.1972
Pentosidine	6	6.1	C17 H26 N6 O4	33.075	378.2016	377.1943
Lonchocarpol B	-	-	C25 H30 O7	33.701	442.1989	441.1916
9-OxoOTrE	-	-	C18 H28 O3	33.827	292.204	291.1967
Abyssinin III	-	-	C25 H28 O6	34.704	424.1884	423.1812
9-OxoODE	1	1.2	C18 H30 O3	36.032	294.2197	293.2124
cis-9,10-Epoxystearic acid	1	1.2	C18 H34 O3	36.708	298.2509	297.2437
Punctaporin B	2	-	C15 H24 O3	41.318	252.1725	251.1653
5-Androstene-3b,16b,17a-triol	3	3.7	C19 H30 O3	41.419	306.2198	305.2127
Heliocide H2	19	19.17	C25 H30 O5	41.532	410.2098	409.2026
Pinolenic Acid	1	1.2	C18 H30 O2	41.695	278.2246	277.2174
Tetrahydrocorticosterone	3	3.2	C21 H34 O4	42.722	350.2458	349.2385
9(Z),11(E)-Conjugated Linoleic Acid	1	1.2	C18 H32 O2	43.774	280.2404	279.2331
Scarlet Red	19	-	C24 H20 N4 O	43.787	380.1651	379.158
methyl 9,10-epoxy-12,15-octadecadienoate	19	-	C19 H32 O3	43.824	308.2351	307.2278
3R-hydroxy-eicosanoic acid	1	1.4	C20 H40 O3	45.779	328.2985	327.2913
2-hydroxy behenic	1	1.4	C22 H44 O3	45.866	356.3298	355.3227
Ro 31-7549	-	-	C24 H22 N4 O2	46.317	398.1756	397.1688
S-Japonin	2	2.5	C19 H28 O3 S	46.33	336.1755	381.1738
α-Linolenic Acid	1	1.2	C18 H30 O2	47.019	278.225	277.2177
6-Deoxotyphasterol	-	-	C28 H50 O3	47.094	434.3757	479.374
5-b-Cholestane-3a ,7a ,12a-triol	3	3.5	C27 H48 O3	47.157	420.3599	419.3527
Androsterone sulfate	3	3.8	C19 H30 O5 S	47.507	370.181	369.1737
5β-Cholestane-3α,7α,12α-triol	3	3.5	C27 H48 O3	47.532	420.3596	419.3525
PS(22:6(4Z,7Z,10Z,13Z,16Z,19Z)/17:2(9Z,12Z))	-	-	C45 H72 N O10 P	48.309	817.4859	816.4789
Phytomonic Acid	-	-	C19 H36 O2	48.585	296.2714	295.2641
PA(20:5(5Z,8Z,11Z,14Z,17Z)/12:0)	-	-	C35 H59 O8 P	49.161	638.3923	637.3853
Piperonyl sulfoxide	19	-	C18 H28 O3 S	49.237	324.1756	383.1895
3'-N-Acetyl-4'-O-(10,12-octadecadienoyl)fusarochromanone	1	1.2	C35 H52 N2 O6	49.537	596.3813	655.3956

Table S1C. EtOAc partition [positive mode]

Compound	Class	Subclass	Molecular Formula	Retention Time (min)	Mass	Product Ions (m/z)
Choline	5	5.2	C5H14NO	1.982	104.1078	104.1072
Isoamyl nitrite	19	19.24	C5 H11 N O2	2.057	117.0791	140.0683
Betaine	6	6.1	C5H12NO2	2.057	118.087	118.0864
Adenine	26	26.1	C5 H5 N5	3.034	135.0546	136.0619
Niacinamide	19	19.25	C6 H6 N2 O	3.986	122.0481	123.0554
3-Pyrimidin-2-yl-2-pyrimidin-2-ylmethyl-Propionic Acid	6	6.2	C12 H12 N4 O2	4.487	244.0969	262.1307
Niazirin	8	8.3	C14 H17 N O5	4.512	279.1113	297.1451
Butamben	20	20.1	C11 H15 N O2	4.537	193.1105	194.1178
D-Alanyl-(R)-lactate	-	-	C6 H11 N O4	4.637	161.0693	184.0585
1,2,3,4-Tetrahydro-b-carboline-1,3-dicarboxylic acid	19	-	C13 H12 N2 O4	5.201	260.08	261.0872
Anhalonidine	-	-	C12 H17 N O3	20.523	223.1213	224.1286
2,5-Dihydro-2,4-dimethyloxazole	19	19.26	C5 H9 N O	20.949	99.0686	100.0759
6Z-Octene-2,4-diynoic acid	-	-	C8 H6 O2	21.099	134.0368	135.044
Asparenyol	11	11.1	C18 H16 O3	21.325	280.1088	303.098
Ser-Ile-OH	4	-	C15 H20 N2 O7	22.427	340.1276	341.1349
Methyl 1-methoxy-1H-indole-3-carboxylate	23	23.1	C11 H11 N O3	22.978	205.0742	206.0815
Oxyquinoline	14	-	C9 H7 N O	23.83	145.0532	146.0605
Dinoseb acetate	22	-	C12 H14 N2 O6	24.031	282.0861	305.0754
N-Hydroxy-L-tryptophan	23	23.1	C11 H12 N2 O3	24.181	220.0852	221.0925
Methyl 2,3-dihydro-3,5-dihydroxy-2-oxo-3-indoleacetic acid	23	23.1	C11 H11 N O5	24.281	237.0643	238.0715
N5-(3,4-Dioxo-1,5-cyclohexadien-1-yl)-L-glutamine	6	6.1	C11 H12 N2 O5	24.419	252.0752	253.0824
Dihydropteroic acid	25	25.1	C14 H14 N6 O3	24.882	314.112	337.101
trans-4,5-Dihydroxy-4,5-dihydropyrene	-	-	C16 H12 O2	24.982	236.0835	259.0726
2,3-dihydro-2-oxo-1H-Benzimidazole-1-propanoic acid	-	-	C10 H10 N2 O3	24.995	206.0698	207.0772
Parathion	19	19.27	C10 H14 N O5 P S	25.083	291.0327	309.0661
Idebenone Metabolite (Benzenebutanoic acid, 2,5-dihydroxy-3,4-dimethoxy-6-methyl-)	-	-	C13 H18 O6	25.096	270.1114	293.1007
HoPhe-His-OH	-	-	C21 H20 N4 O6	25.183	424.1394	425.1467
3-O-Methylniveusin A	2	2.3	C21 H28 O8	25.183	408.1777	409.1849
2-(diethylamino)-4'-hydroxy-Propiophenone	-	-	C13 H19 N O2	25.334	221.1421	222.1494
methyl 9-hydroperoxy-10,12,13,15-bisepidioxy-16E-octadecenoate	-	-	C19 H32 O8	25.396	388.2105	411.1999
Sonchuionoside C	2	2.4	C19 H30 O8	25.471	386.1948	409.184
Pro Trp Gly	4	-	C18 H22 N4 O4	25.496	358.1638	381.1531
Alpha-Pyrrolidinopropiophenone	-	-	C13 H17 N O	25.534	203.1314	204.1387
Methyl 1-(methylsulfinyl)propyl disulfide	19	-	C5 H12 O S3	25.659	184.0056	185.0128
1,4-Benzodioxin-2(3H)-one	19	19.28	C8 H6 O3	25.822	150.0321	151.0392
trans-p-Coumaric acid 4-glucoside	-	-	C15 H18 O8	25.96	326.1011	349.0903
4,5-seco-Dopa	6	6.1	C9 H11 N O6	25.985	229.0593	252.0484
Glucosyl (2E,6E,10x)-10,11-dihydroxy-2,6-farnesadienoate	2	2.4	C21 H36 O9	26.085	432.2363	455.2256
Trp-P-1	4	-	C13 H13 N3	26.198	211.1117	212.119
Anatoxin a(s)	19	-	C7 H17 N4 O4 P	26.336	252.098	270.1317
trans-Grandmarin	24	24.2	C15 H16 O6	26.398	292.0953	315.0845
**Compound**	**Class**	**Subclass**	**Molecular Formula**	**Retention Time (min)**	**Mass**	**Product Ions (m/z)**
Pisumionoside	2	2.4	C19 H32 O9	26.624	404.2052	427.1944
2-Phenylethyl beta-D-glucopyranoside	8	8.3	C14 H20 O6	26.661	284.1264	307.1161

(R)-1-O-b-D-glucopyranosyl-1,3-octanediol	1	1.8	C14 H28 O7	26.762	308.1843	331.1735
3-Methylbutyl 2-furanbutanoate	1	1.9	C13 H20 O3	26.837	224.1416	247.1309
Niazirinin	8	8.3	C16 H19 N O6	26.962	321.1219	344.1114
Mecarbinzid	-	-	C13 H16 N4 O3 S	26.962	308.0949	309.1017
2,5-Diamino-6-hydroxy-4-(5'-phosphoribosylamino)-pyrimidine	-	-	C9 H16 N5 O8 P	27.012	353.0734	354.0805
5-Hydroxythalidomide	19	19.29	C13 H10 N2 O5	27.138	274.0599	292.0938
Hexahydro-6,7-dihydroxy-5-(hydroxymethyl)-3-(2-hydroxyphenyl)-2H-pyrano[2,3-d]oxazol-2-one	21	21.4	C13 H15 N O7	27.263	297.0847	298.0919
Disuccinimidyl Suberate	6	6.3	C16 H20 N2 O8	27.288	368.1229	391.1122
Verimol C	11	11.1	C18 H20 O4	27.325	300.1356	323.1248
trans-3-Hydroxycotinine glucuronide	8	8.4	C16 H20 N2 O8	27.388	368.1228	369.1299
8-Acetylegelolide	19	-	C16 H20 O6	27.438	308.1269	331.1161
Dopaquinone	6	6.1	C9 H9 N O4	27.463	195.0539	196.0612
3,5-Pyridinedicarboxylic acid, 2-(hydroxymethyl)-6-methyl-4-(3-nitrophenyl)-, 5-(2-hydroxyethyl) est	-	-	C17 H16 N2 O8	27.588	376.0918	394.1256
5-Megastigmen-7-yne-3,9-diol 9-glucoside	1	1.8	C19 H30 O7	27.589	370.2003	393.1896
alpha-Peroxyachifolide	2	2.3	C20 H24 O7	27.664	376.1531	399.1423
(E)-2-Glucosyl-3,4',5-trihydroxystilbene	7	7.1	C20 H22 O8	27.739	390.1323	413.1216
4-Hydroxynornantenine	19	-	C19 H19 N O5	27.927	341.1257	364.1147
7,8-Didemethyl-8-hydroxy-5-deazariboflavin	-	-	C16 H17 N3 O7	27.927	363.1075	386.0966
Viguiestenin	-	-	C21 H28 O7	27.952	392.1824	393.1896
Isobutyl 2-furanpropionate	1	1.9	C11 H16 O3	28.04	196.1105	219.0998
DHAP(8:0)	8	8.2	C11 H21 O7 P	28.202	296.1022	319.0915
Gly Arg Tyr	4	-	C17 H26 N6 O5	28.202	394.1983	395.2056
3-(3,4-Methylene dioxyphenyl)propenal	19	-	C10 H8 O3	28.34	176.048	177.0553
N2-(gamma-Glutamyl)-4-carboxyphenylhydrazine	6	6.1	C12 H15 N3 O5	28.49	281.1018	304.0908
Felbamate monocarboxylate	20	-	C10 H11 N O4	28.503	209.0693	210.0767
2,3-dinor Thromboxane B1	-	-	C18 H32 O6	28.528	344.2205	367.2099
4-Hydroxycoumarin	24	24.1	C9 H6 O3	28.541	162.0324	163.0398
3-Hydroxychavicol 1-glucoside	8	8.3	C15 H20 O7	28.666	312.1203	335.1106
Isoamyl p-anisate	20	20.1	C13 H18 O3	28.691	222.126	223.1333
1,3,5,8-Tetrahydroxy-6-methoxy-2-methylanthraquinone	19	19.17	C16 H12 O7	28.854	316.0575	334.0914
N-(1-Deoxy-1-fructosyl)methionine	1	1.8	C11 H21 N O7 S	28.854	311.1025	312.1096
2-Ethoxy-5-(1-propenyl)phenol	17	-	C11 H14 O2	29.092	178.1	179.1073
2-(3,4-Dihydroxyphenylethyl)-6-epi-elenaiate	21	21.5	C19 H22 O8	29.142	378.1307	379.1379
1,2-beta-D-Glucuronosyl-D-glucuronate	-	-	C12 H18 O13	29.192	370.0744	371.0818
Cys Glu Phe	4	-	C17 H23 N3 O6 S	29.242	397.1317	415.1654
(-)-11-Hydroxy-9,15,16-trioxooctadecanoic acid	1	1.4	C18 H30 O6	29.367	342.2052	365.1945
5,6-Epoxy-5,6-dihydro-10'-apo-b,y-carotene-3,10'-diol	2	2.8	C27 H38 O3	29.442	410.2813	433.2706
Leu His Asn	4	-	C16 H26 N6 O5	29.468	382.1971	383.2043
Polyethylene, oxidized	19	19.23	C12 H20 O5	29.568	244.13	267.1209
1,8-Dihydroxy-3,5-dimethoxy-2-prenylxanthone	9	9.1	C20 H20 O6	29.969	356.1259	379.1141
**Compound**	**Class**	**Subclass**	**Molecular Formula**	**Retention Time (min)**	**Mass**	**Product Ions (m/z)**
PA(19:1(9Z)/0:0)	-	-	C22 H43 O7 P	30.094	450.2737	451.2812
(±)-Clausenamide	19	19.30	C18 H19 N O3	30.144	297.136	320.1252
Tryptophyl-Aspartate	6	6.1	C15 H17 N3 O5	30.194	319.1178	342.107

Casimiroin	14	14.5	C12 H11 N O4	30.294	233.0691	251.1033
epi-Tulipinolide diepoxide	2	2.3	C17 H22 O6	30.394	322.1402	323.1476
(-)-Bisdechlorogeodin	-	-	C17 H14 O7	30.445	330.0731	348.1069
4R,5R,6S-Trihydroxy-2-hydroxymethyl-2-cyclohexen-1-one 6-(2-hydroxy-6-methylbenzoate)	20	20.1	C15 H16 O7	30.47	308.0909	326.1247
Galactitol 1-phosphate	8	8.3	C6 H15 O9 P	30.633	262.0465	280.0802
Erythronolide B	-	-	C21 H38 O7	30.695	402.2619	425.2514
6-Azaequilenin	-	-	C17 H17 N O2	30.77	267.1252	290.1144
Propyl 1-(propylsulfinyl)propyl disulfide	19	-	C9 H20 O S3	30.896	240.0684	241.0751
Hulupinic acid	19	-	C15 H20 O4	30.921	264.1368	287.1261
2-Methyl-3-(2-pentenyl)-2-cyclopenten-1-one	8	8.2	C11 H16 O	30.996	164.1203	165.1276
(3R,8E)-3-Hydroxy-5,8-megastigmadien-7-one	8	8.2	C13 H20 O2	30.996	208.1467	209.154
Methyl dioxindole-3-acetate	23	23.2	C11 H11 N O4	31.096	221.0689	222.0763
p-Hydroxyphenytoin glucuronide	19	19.31	C21 H20 N2 O9	31.147	444.1156	445.1227
Italipyrone	20	20.6	C22 H24 O7	31.196	400.1518	423.1409
(+)-Ligballinol	19	-	C18 H18 O4	31.334	298.1203	321.1097
7a-Hydroxy-O-carbamoyl-deacetylcephalosporin C	-	-	C15 H20 N4 O9 S	31.547	432.0964	239.0372
Coumarin	24	-	C9 H6 O2	31.648	146.0375	147.0448
(9R,10S,12Z)-9,10-Dihydroxy-8-oxo-12-octadecenoic acid	1	1.2	C18 H32 O5	31.76	328.2262	351.2154
Glu Thr Thr	4	-	C13 H23 N3 O8	31.823	349.1478	367.1817
Pyridine-2-azo-p-dimethylaniline	-	-	C13 H14 N4	32.023	226.1214	249.1107
3,5-Pyridinedicarboxylic acid, 1,4-dihydro-2,6-dimethyl-4-(3-nitrophenyl)-, 2-hydroxyethyl methyl es	-	-	C18 H20 N2 O7	32.174	376.1283	399.1175
2,3-dinor Prostaglandin E1	1	1.5	C18 H30 O5	32.249	326.2103	349.1996
Alfuzosin	18	18.1	C19 H27 N5 O4	32.499	389.2077	407.2415
9S,10S,11R-trihydroxy-12Z-octadecenoic acid	-	-	C18 H34 O5	32.562	330.2418	353.2311
4-Epitetracycline	13	-	C22 H24 N2 O8	32.8	444.152	445.1593
(3b,6b,8a,12a)-8,12-Epoxy-7(11)-eremophilene-6,8,12-trimethoxy-3-ol	19	-	C18 H30 O5	32.988	326.2098	349.1991
3-(N-Maleimidopropionyl)-biocytin	19	19.32	C23 H33 N5 O7 S	33	523.2091	541.2419
10-hydroxy-hexadecan-1,16-dioic acid	-	-	C16 H30 O5	33.076	302.2099	325.1991
Oenanthoside A	8	8.3	C16 H20 O8	33.151	340.1169	363.1061
trans-Cinnamic acid	12	12.3	C9 H8 O2	33.201	148.0531	149.0603
Sulfapyridine	20	20.5	C11 H11 N3 O2 S	33.251	249.0577	267.0913
Furcelleran	12	12.1	C31 H27 N O4	33.301	477.1933	495.2271
Lactapiperanol D	2	2.2	C18 H28 O5	33.376	324.1944	347.1837
16-Methyl-epi-nigakihemiacetal B	2	2.3	C23 H32 O6	33.401	404.2187	405.2253
2,3-dinor-8-iso-PGF2a	-	-	C18 H30 O5	33.439	326.2095	349.1988
Palmitic amide	1	1.1	C16 H33 N O	33.577	255.2571	256.2645
C16 Sphinganine	5	5.1	C16 H35 N O2	33.602	273.2679	296.2572
15-keto-Prostaglandin E2	1	1.5	C20 H30 O5	33.702	350.2082	351.2151
Phytosphingosine	5	5.1	C18 H39 N O3	33.802	317.2938	318.3012
2-Hydroxyhexadecanoic acid	-	-	C16 H32 O3	33.915	272.2362	290.2701
Sulfadimidine	20	20.5	C12 H14 N4 O2 S	34.002	278.0841	279.0914
13S-HpOTrE	1	1.2	C18 H30 O4	34.078	310.2154	333.2047
**Compound**	**Class**	**Subclass**	**Molecular Formula**	**Retention Time (min)**	**Mass**	**Product Ions (m/z)**
Imiquimod	14	14.5	C14 H16 N4	34.116	240.1371	263.1264
Deuteroporphyrin IX	10	10.1	C30 H30 N4 O4	34.278	510.2263	533.2153
C17 Sphinganine	5	5.1	C17 H37 N O2	34.354	287.2835	288.2908
Coriandrone D	9	9.2	C18 H24 O7	34.403	352.1533	375.1426

Acetyl Tyrosine Ethyl Ester	-	-	C13 H17 N O4	34.454	251.1165	252.1238
2-Hydroxyestradiol	3	3.9	C18 H24 O3	34.479	288.1731	311.1624
Rugosinone	-	-	C19 H15 N O6	34.504	353.0882	371.1221
Citrusinine I	14	14.4	C16 H15 N O5	34.604	301.0949	319.1291
2-Carboxy-4-dodecanolide	19	19.33	C13 H22 O4	34.667	242.1527	265.142
Letrozole	19	19.4	C17 H11 N5	34.855	285.1015	286.1088
3,3',4,4'-Tetrahydroxy-5,5'-diisopropyl-2,2'-dimethylbiphenyl	19	19.5	C20 H26 O4	34.98	330.1821	331.1892
(9Z,11E,13E,15Z)-4-Oxo-9,11,13,15-octadecatetraenoic acid	1	1.2	C18 H26 O3	35.005	290.1895	291.1967
Nepetaside	2	2.4	C16 H26 O8	35.092	346.1617	347.169
Annoglabasin C	2	2.2	C23 H34 O6	35.105	406.2344	407.2415
Dihydrocapsiate	21	21.1	C18 H28 O4	35.305	308.1995	331.1888
7E,10-undecadien-4-olide	-	-	C11 H16 O2	35.305	180.1155	203.1048
Thyrotropin releasing hormone	-	-	C16 H22 N6 O4	35.506	362.1698	363.1787
8-iso Prostaglandin F1β	1	1.5	C20 H36 O5	35.556	356.2572	379.2466
Granisetron	19	19.2	C18 H24 N4 O	35.706	312.1951	335.1844
6-Hydroxyenterodiol	19	19.34	C18 H22 O5	35.757	318.1476	341.1367
15-Deacetylcalonectrin			C17 H24 O5	35.857	308.1636	331.153
16-phenyl-tetranor-PGE2			C22 H28 O5	35.932	372.1954	395.1846
11β-Prostaglandin F1β	1	1.5	C20 H36 O5	35.982	356.2565	379.2461
2',4'-Dihydroxy-7-methoxy-8-prenylflavan	17	17.1	C21 H24 O4	36.032	340.1669	363.156
Dinor Iloprost	1	1.5	C20 H28 O4	36.082	332.1977	333.2047
Bicuculline (+)	19	-	C20 H17 N O6	36.107	367.105	385.139
12-Hydroxydihydrochelirubine	-	-	C21 H17 N O6	36.183	379.1065	397.1404
Bis(2-methylpropanoyloxy)-9,10-epoxy-p-mentha-1,3,5-triene	22	-	C18 H24 O5	36.22	320.1632	343.1524
3-methoxy Prostaglandin F1α	1	1.5	C21 H38 O6	36.458	386.2675	409.2567
9,12-dimethoxy-13-hydroxy-10-octadecenoic acid	-	-	C20 H38 O5	36.659	358.2726	381.262
Thr Met Thr	4	-	C13 H25 N3 O6 S	36.884	351.1471	369.1812
Citronellyl alpha-toluate	1	1.3	C18 H26 O2	37.009	274.194	275.2013
beta-Zearalanol	16	-	C18 H26 O5	37.034	322.1788	345.1681
(9Z,11S,16S)-1-Acetoxy-9,17-octadecadiene-12,14-diyne-11,16-diol	1	7	C20 H28 O4	37.16	332.1979	333.205
Rosoxacin	14	14.1	C17 H14 N2 O3	37.185	294.1013	295.1086
Lys Ser Asp	4	-	C13 H24 N4 O7	37.197	348.1641	349.171
Maculosin	6	6.1	C14 H16 N2 O3	37.21	260.1167	261.124
15-methyl-15(S)-PGE1	-	-	C21 H36 O5	37.598	368.2572	391.2466
Horhammericine	-	-	C21 H24 N2 O4	37.836	368.1729	369.181
9-hydroperoxy-10E,12,15Z-octadecatrienoic acid	1	1.2	C18 H30 O4	37.911	310.215	333.2043
4,6'-Epoxyorotiniflavan-4-ol	-	-	C26 H28 O6	38.012	436.1881	437.1955
(ent-16betaOH)-16,17-Dihydroxy-9(11)-kauren-19-oic acid	7	-	C20 H30 O4	38.074	334.2136	335.2209
(+)-Sceletium A4	-	-	C20 H24 N2 O2	38.137	324.1851	325.1924
Asp Thr Val	4	-	C13 H23 N3 O7	38.337	333.1529	351.1867
3,4-Dimethyl-5-pentyl-2-furanheptanoic acid	1	1.4	C18 H30 O3	38.412	294.2204	295.2277
15-methyl-15S-PGE2	-	-	C21 H34 O5	38.525	366.2419	389.231
9-hydroxy-13-oxo-10-octadecenoic acid	1	1.2	C18 H32 O4	38.676	312.2313	335.2205
**Compound**	**Class**	**Subclass**	**Molecular Formula**	**Retention Time (min)**	**Mass**	**Product Ions (m/z)**
3b-Hydroxy-6b-tigloyloxy-7(11)-eremophilen-12,8b-olide	2	2.3	C20 H28 O5	38.713	348.1948	371.184
8-HpODE	1	1.2	C18 H32 O4	38.864	312.2308	335.2201
Piperoic acid	2	2.5	C22 H30 O4	38.926	358.2155	381.2048
1-Naphthylacetylspermine	-	-	C22 H34 N4 O	39.089	370.2728	393.2621

Piperochromanoic acid	2	2.5	C22 H28 O4	39.139	356.1998	379.1891
Lyciumoside III	2	2.4	C32 H56 O13	39.239	648.3697	347.1743
Gingerglycolipid A	19	19.35	C33 H56 O14	39.264	676.366	677.3735
[7]-Paradol	21	21.1	C18 H28 O3	39.34	292.2048	315.1941
3alpha,12alpha-Dihydroxy-5beta-chola-8,14-dien-24-oic Acid	-	-	C24 H36 O4	39.39	388.2595	389.2669
Stearidonic Acid	1	1.2	C18 H28 O2	39.59	276.2101	277.2173
13-OxoODE	1	1.2	C18 H30 O3	39.69	294.2205	317.2099
(S)-3-Octanol glucoside	1	1.8	C14 H28 O6	39.715	292.1895	315.1788
18-Hydroxycorticosterone	3	3.2	C21 H30 O5	40.367	362.2084	363.2157
Ganoderic acid eta	2	2.6	C30 H44 O8	40.417	532.3048	555.294
18:1(5Z)(9Me,13Me,17Me)	-	-	C21 H40 O2	40.43	324.3036	342.3375
PI(16:1(9Z)/12:0)	-	-	C37 H69 O13 P	40.492	752.4464	377.2301
Auxin b	2	2.1	C18 H30 O4	40.555	310.2156	333.2046
Methyl cis-p-coumarate 3-(3,7-dimethyl-2,6-octadienyl)	12	12.1	C20 H26 O3	40.68	314.1874	315.1946
9-Docosene	19	19.7	C22 H44	40.793	308.3457	326.3795
8-iso Prostaglandin E2 isopropyl ester			C23 H38 O5	40.993	394.2701	395.2775
Acetylenic acids; 17-Octadecen-9-ynoic acid	1	1.4	C18 H30 O2	41.181	278.2257	279.233
2-Stearyl citrate	6	6.4	C24 H44 O7	41.67	444.3074	445.3148
Arg Ile Ile	4	-	C18 H36 N6 O4	41.845	400.2811	401.2884
Lucidenic acid A	2	2.5	C27 H38 O6	41.908	458.2679	481.2571
Lys Val Trp	4	-	C22 H33 N5 O4	42.321	431.2537	449.2875
13,16-Octadecadiynoic acid	1	1.4	C18 H28 O2	42.459	276.21	277.2171
Arg Pro Phe	4	-	C20 H30 N6 O4	42.622	418.2336	419.2409
Lucidenic acid N	2	2.5	C27 H40 O6	43.01	460.2833	483.2724
2,2-Dibutyl-3-(4-methoxyphenyl)-4-methyl-2H-1-benzopyran-7-ol acetate	-	-	C27 H34 O4	43.123	422.2463	445.2358
Diisobutyl phthalate	20	20.1	C16 H22 O4	43.324	278.1527	301.142
Insignin A	-	-	C21 H32 O5	43.474	364.2261	387.2153
18-Oxooleate	-	-	C18 H32 O3	43.712	296.2361	319.2252
2,9-Bis(3-methyl-2E-pentenoyl)-2b,9a-dihydroxy-4Z,10(14)-oplopadien-3-one	-	-	C27 H38 O5	43.787	442.273	465.2623
(25S)-5alpha-cholestan-3beta,4beta,6alpha,8beta,15alpha,16beta,26-heptol	-	-	C27 H48 O7	44.163	484.3406	507.33
Leu Trp Lys	4	-	C23 H35 N5 O4	44.2	445.2703	463.3042
10-F2-dihomo-IsoP	-	-	C23 H40 O5	44.301	396.2884	419.2779
Montanol	1	1.4	C21 H36 O4	44.363	352.2623	375.2515
Deoxycorticosterone	3	3.2	C21 H30 O3	44.526	330.2179	331.2253
6-HpOME(7E)	-	-	C18 H34 O4	44.877	314.2469	337.2361
Hexyl heptanoate	1	1.9	C35 H34 N4 O8	44.902	638.2387	639.246
Trp Val Val	4	-	C21 H30 N4 O4	44.977	402.227	425.2164
Kammogenin	-	-	C27 H40 O5	45.14	444.2885	467.2776
Polidocanol	8	8.1	C30 H62 O10	45.178	582.4344	605.4236
4'-O-Methylneobavaisoflavone 7-O-(2''-p-coumaroylglucoside)	-	-	C36 H36 O11	45.328	644.2244	645.2318
Glyceryl lactooleate	1	1.9	C24 H44 O6	45.641	428.313	429.3204
17,20-dimethyl Prostaglandin F1α	1	1.5	C22 H40 O5	45.754	384.2864	385.2938
Thromboxanoic acid skeleton	-	-	C20 H36 O4	45.754	340.2598	341.2671
**Compound**	**Class**	**Subclass**	**Molecular Formula**	**Retention Time (min)**	**Mass**	**Product Ions (m/z)**
12(13)-EpOME	1	1.4	C18 H32 O3	45.804	296.2337	297.241
Phaeophorbide b	10	10.2	C35 H34 N4 O6	45.904	606.2489	607.256
AT-56	-	-	C25 H27 N5	46.105	397.2264	398.2338
3,6-Epoxy-5,5',6,6'-tetrahydro-b,b-carotene-3',5,5',6'-tetrol	2	2.10	C40 H58 O5	46.23	618.4309	619.4386

Anandamide (20:2, n-6)	-	-	C22 H41 N O2	46.255	351.3141	374.3034
PG(6:0/6:0)	-	-	C18 H35 O10 P	46.405	442.1981	443.2057
Cetiedil	19	-	C20 H31 N O2 S	46.631	349.2061	367.2399
PI(13:0/0:0)	-	-	C22 H43 O12 P	46.881	530.2501	531.2577
Desglucocheirotoxol	3	3.3	C29 H44 O10	47.007	552.2947	575.2843
Terbucarb	-	-	C17 H27 N O2	47.031	277.2049	278.212
9-Pentacosene	19	19.36	C25 H50	47.482	350.3914	368.4252
Harderoporphyrin	10	10.1	C35 H36 N4 O6	47.545	608.265	609.2725
Kanokoside D	2	2.4	C27 H44 O16	47.633	624.26	625.2675
Oleamide	1	1.1	C18 H35 N O	47.708	281.2727	282.28
13E-Docosenamide	1	1.1	C22 H43 N O	47.821	337.3354	338.3426
Pheophorbide a	-	-	C35 H36 N4 O5	48.498	592.2706	593.2782
1α,25-dihydroxy-22-oxavitamin D3 3-hemiglutarate/ 1α,25-dihydroxy-22-oxacholecalciferol 3-hemiglutarate	-	-	C31 H48 O7	48.51	532.3382	533.3456
3alpha,7alpha,12alpha,16alpha-Tetrahydroxy-5beta-cholestan-26-oic acid	-	-	C27 H46 O6	48.635	466.33	489.3192
23-demethylgorgosterol	-	-	C29 H48 O	48.835	412.3708	413.3782
Haplophytine	-	-	C37 H40 N4 O7	49.537	652.289	653.2962

Table S1D. EtOAc partition [negative mode]

**Compound**	**Class**	**Subclass**	**Molecular Formula**	**Retention Time (min)**	**Mass**	**Product Ions (m/z)**
4-Oxoglutaramate	19	19.18	C5 H7 N O4	2.694	145.0372	144.0299
5-Methoxysalicylic acid	20	20.1	C8 H8 O4	9.81	168.0421	167.0349
2,4,6-Trimethyl-3,5-dinitrobenzonitrile	-	-	C10 H9 N3 O4	10.261	235.0588	294.0727

2,5-Dihydroxybenzaldehyde	20	20.2	C7 H6 O3	12.215	138.0318	137.0245
Benzyl O-[arabinofuranosyl-(1->6)-glucoside]	8	8.3	C18 H26 O10	12.842	402.1522	401.145
4-Acetoxyphenol	22	-	C8 H8 O3	13.105	152.0473	151.0401
Esculetin	24	24.1	C9 H6 O4	13.118	178.0268	177.0196
5a,6a-Epoxy-7E-megastigmene-3b,9e-diol 9-glucoside	1	1.8	C19 H32 O8	13.243	388.2093	433.2076
Dihydroxyphenylacetic acid	19	19.19	C8 H8 O4	13.293	168.0422	167.0349
Dihydroconiferin	8	8.3	C16 H24 O8	13.343	344.1467	403.1606
3-(3-Furanyl)-2-methyl-2-propenal	19	-	C8 H8 O2	13.368	136.0522	135.0449
Dihydromelilotoside	8	8.3	C15 H20 O8	13.418	328.1153	327.1082
Vanillylmandelic acid	21	21.1	C9 H10 O5	13.518	198.0525	197.0452
Dihydroroseoside	1	1.8	C19 H32 O8	13.944	388.2092	433.2074
2-Hydroxycinnamic acid	12	12.2	C9 H8 O3	13.969	164.0471	163.0398
Samin	19	-	C13 H14 O5	14.02	250.0838	249.0769
Picrotoxinin	19	-	C15 H16 O6	14.145	292.0942	291.0868
3,4-Dihydroxybenzaldehyde	8	8.2	C7 H6 O3	14.17	138.0315	137.0242
Bacteriocin 28b	6	6.1	C12 H11 N3 O2	14.245	229.0849	274.0831
4-Hydroxybenzaldehyde	8	8.2	C7 H6 O2	14.32	122.0369	121.0296
Corchoionol C 9-glucoside	1	1.8	C19 H30 O8	14.345	386.1937	431.1917
Phenylethyl primeveroside	8	8.3	C19 H28 O10	14.37	416.1677	415.1605
(-)-11-hydroxy-9,10-dihydrojasmonic acid 11-beta-D-glucoside	-	-	C18 H30 O9	14.621	390.1883	449.2022
Rutin	17	17.2	C27 H30 O16	14.922	610.1522	609.1448
5-(3,4-Dihydroxyphenyl)-5-ethylbarbituric acid	-	-	C12 H12 N2 O5	15.022	264.0744	323.0882
3-Hydroxycoumarin	24	24.1	C9 H6 O3	15.047	162.0315	161.0242
Methyl 2,6-dihydroxy-4-quinolinecarboxylate	14	14.3	C11 H9 N O4	15.347	219.0529	218.0457
ethyl 6,7-dimethoxy-4-oxo-2,3-dihydro-1H-naphthalene-2-carboxylate	-	-	C15 H18 O5	15.423	278.1151	323.1134
Vitexin 4'-O-galactoside	17	17.2	C27 H30 O15	15.523	594.1574	593.1502
Blumenol C glucoside	1	1.8	C19 H32 O7	15.623	372.2144	417.2127
Propanoic acid, 2-hydroxy-3-[2-(2-propenyloxy)phenoxy]-	-	-	C12 H14 O5	15.748	238.0839	237.0767
6-C-Xylopyranosyl-8-C-glucopyranosylchrysoeriol	-	-	C27 H30 O15	15.824	594.1571	593.1498
Ethyl 7-epi-12-hydroxyjasmonate glucoside	1	1.8	C20 H32 O9	16.036	416.2043	415.197
Ferulic acid	12	12.2	C10 H10 O4	16.074	194.0579	193.0506
2,6-Dihydroxynicotinate	19	19.14	C6 H5 N O4	16.275	155.0218	154.0146
9-Hydroxy-7-megastigmen-3-one glucoside	1	1.8	C19 H32 O7	16.5	372.2151	417.2134
Scytalone	-	-	C10 H10 O4	16.55	194.0579	193.0507
Phenylacetonitrile	8	-	C8 H7 N	17.214	117.0579	116.0507
3-Hydroxyisoheptanoic acid	19	19.20	C7 H14 O3	17.227	146.0941	145.0869
Methyl N-(a-methylbutyryl)glycine	1	1.4	C9 H16 O4	17.427	188.1051	187.0979
4-formyl Indole	-	-	C9 H7 N O	17.527	145.0531	144.0459
m-Coumaric acid	12	12.2	C9 H8 O3	17.803	164.0475	163.0402
xi-2,3-Octadiene-5,7-diyn-1-ol	1	1.7	C8 H6 O	17.828	118.0418	117.0346

**Compound**	**Class**	**Subclass**	**Molecular Formula**	**Retention Time (min)**	**Mass**	**Product Ions (m/z)**
(-)-11-hydroxy-9,10-dihydrojasmonic acid	1	1.2	C12 H20 O4	17.953	228.1361	227.1289
Naproxen glucuronide	8	8.3	C20 H22 O9	17.978	406.126	405.1188
2-Phthalimidoglutaramic acid	1	1.4	C13 H12 N2 O5	18.179	276.0743	321.0724
Grandisine III	14	14.4	C15 H13 N O5	18.228	287.0789	332.0772

Homovanillic acid	21	21.1	C9 H10 O4	18.354	182.0577	181.0504
N-(3-oxo-octanoyl)-homoserine lactone	6	6.1	C12 H19 N O4	18.454	241.1311	240.124
Asp-Gly-OH	6	6.1	C11 H10 N2 O8	18.655	298.0452	297.0379
(3S,7E,9S)-9-Hydroxy-4,7-megastigmadien-3-one 9-glucoside	1	1.8	C19 H30 O7	18.93	370.1986	415.1968
4-Nitrophenol	21	21.2	C6 H5 N O3	19.181	139.0273	138.02
Mundoserone	-	-	C19 H18 O6	19.356	342.11	341.1028
Abscisic Acid (cis,trans)	2	2.5	C15 H20 O4	19.432	264.1359	263.1286
1-(3,4-Dihydroxyphenyl)-7-(4-hydroxy-3-methoxyphenyl)-1,6-heptadiene-3,5-dione	19	19.3	C20 H18 O6	19.444	354.1119	353.1048
2-Methyl-1-phenyl-2-propanyl butyrate	8	8.3	C14 H20 O2	19.456	220.1459	219.1386
5,7,4'-Trihydroxy-3'-methoxyflavanone 4'-O-isobutyrate	-	-	C20 H20 O7	19.532	372.1204	371.1132
Sebacic acid	1	1.4	C10 H18 O4	19.632	202.1202	201.1129
Distichonic acid B	6	6.1	C10 H18 N2 O8	20.884	294.1076	223.0949
Tuberonic acid	6	6.1	C12 H18 O4	20.885	226.1207	293.1002
Ethoprop	19	-	C8 H19 O2 P S2	21.16	242.0563	225.1134
His Ile Gln	4	-	C17 H28 N6 O5	21.361	396.2122	241.049
9,12,13-trihydroxy-10,15-octadecadienoic acid	1	1.4	C18 H32 O5	21.636	328.2251	327.2179
2,3-Dinor-11b-PGF2a	1	1.5	C18 H30 O5	21.674	326.2092	325.202
Amlexanox	9	9.1	C16 H14 N2 O4	21.862	298.0947	269.1028
Aspidinol	21	21.3	C12 H16 O4	22.388	224.1048	297.0879
11,12,13-trihydroxy-9-octadecenoic acid	1	1.4	C18 H34 O5	22.438	330.241	223.0975
7-Ethyl-2R-methyl-1,6-dioxaspiro[4.5]decane	1	1.4	C11 H20 O2	22.952	184.1465	329.2337
9-hydroxy-hexadecan-1,16-dioic acid	-	-	C16 H30 O5	23.064	302.2093	183.1392
Traumatic Acid	1	1.4	C12 H20 O4	23.127	228.1361	301.2022
4'-Hydroxyfenoprofen	20	20.4	C15 H14 O4	23.34	258.0891	227.1289
3,12-dihydroxy palmitic acid	1	1.4	C16 H32 O4	23.365	288.2298	257.0818
Isoplumbagin	19	19.21	C11 H8 O3	23.39	188.0471	287.2226
3-Methylindolepyruvate	19	19.22	C12 H11 N O3	23.503	217.0738	187.0398
N-methyl-Gabapentin	6	6.1	C10 H19 N O2	23.766	185.1414	184.1341
3,7-Dimethyl-2E,6-octadienyl acetate	-	-	C12 H20 O2	24.004	196.1464	195.1391
(E)-2-Hexenyl (E)-7,9-decadienoate	-	-	C16 H26 O2	24.443	250.1933	309.2069
Gibberellin A65	2	2.2	C20 H26 O6	24.793	362.1734	361.1661
5-Hydroxy-4-methoxy-3-methyl-2,6-canthinedione	11	-	C16 H12 N2 O4	25.345	296.0795	295.0722
5-O-Methylembelin	2	2.7	C18 H28 O4	25.495	308.1991	307.1918
Methylgingerol	8	8.2	C18 H28 O4	25.946	308.1984	307.1911
3-(2-Hydroxyphenyl)propionic acid	19	-	C9 H10 O3	26.196	166.0629	165.0556
Lagochilin	3	3.7	C20 H36 O5	26.46	356.2558	355.2486
Mangostenone B	9	9.1	C28 H30 O6	26.547	462.2033	507.2015
Methylprednisone	3	3.2	C22 H28 O5	26.648	372.1935	371.1864
Inulicin	2	2.3	C17 H24 O5	26.71	308.1622	307.1549
Gibberellin A3	2	2.2	C19 H22 O6	27.048	346.1416	345.1343

Table S2. List of compounds identified in *R. nasutus* callus extract by UHPLC-QToF-MS analysis

Table S2A. Hexane partition [positive mode]

**Compound**	**Class**	**Subclass**	**Molecular Formula**	**Retention Time (min)**	**Mass**	**Product Ions (m/z)**
Theophylline	26	26.1	C7 H8 N4 O2	2.012	180.0644	203.0536

Anhalonidine	-	-	C12 H17 N O3	2.714	223.1218	224.1291
Dikegulac	-	-	C12 H18 O7	3.265	274.1047	275.1121
Methyl red	19	-	C15 H15 N3 O2	6.422	269.1176	270.1249
Sudan I	29	29.1	C16 H12 N2 O	10.406	248.0962	266.13
PE-Cer(d14:2(4E,6E)/ 20:1(11Z)(2OH))	-	-	C36 H69 N2 O7 P	16.469	672.4874	359.233
Pentyl heptanoate	1	1.9	C12 H24 O2	17.021	200.1786	218.2124
Desmethylergometrine	-	-	C18 H21 N3 O2	17.346	311.1647	312.172
Sapidolide A	31	31.1	C14 H18 O5	17.622	266.1146	267.1217
Asn Arg Ala	4	-	C13 H25 N7 O5	19.126	359.191	382.18
Citreovirenone	21	21.1	C14 H16 O4	20.729	248.1041	249.1113
4-Ethyl-2-heptylthiazole	32	32.1	C12 H21 N S	21.456	211.1403	234.1293
His Glu Lys	4	-	C17 H28 N6 O6	21.531	412.2066	413.214
Bisbynin	19	-	C15 H22 O5	21.656	282.1454	283.1527
Ser Arg Pro	4	-	C14 H26 N6 O5	21.681	358.1979	359.2053
Indirubin-3'-monoxime	23	23.3	C16 H11 N3 O2	22.684	277.0839	278.0912
(4E,8E,10E-d18:3) sphingosine	-	-	C18 H33 N O2	24.262	295.2504	296.2578
C16 Sphinganine	5	5.1	C16 H35 N O2	24.487	273.2674	296.2566
3-Oxopregn-4-ene-20beta-carboxaldehyde dioxime	-	-	C22 H34 N2 O2	25.026	358.2635	359.2708
2,3-dinor Prostaglandin E1	1	1.5	C18 H30 O5	25.527	326.2097	349.1989
Gibberellin A97	2	2.2	C20 H28 O6	25.715	364.1879	365.1948
2,3-dinor Thromboxane B1	1	-	C18 H32 O6	26.166	344.2206	367.2099
(-)-11-Hydroxy-9,15,16-trioxooctadecanoic acid	1	1.4	C18 H30 O6	26.279	342.2051	365.1944
Sodium glycocholate	3	3.1	C26 H43 N O6	26.391	465.3092	466.3166
Geranyl acetoacetate	1	-	C18 H32 O6	26.467	344.2205	367.2097
Corrinoid	-	-	C14 H22 O3	26.542	238.158	261.1472
6,8-Dihydroxy-1,7-diprenylxanthone-2-carboxylic acid	9	9.1	C24 H24 O6	26.98	408.1569	409.1644
6-Epi-7-isocucurbic acid glucoside	1	1.8	C18 H30 O8	27.043	374.1949	397.1841
Melochinone	-	-	C22 H21 N O2	27.419	331.1569	349.1907
(9Z,11S,16S)-1-Acetoxy-9,17-octadecadiene-12,14-diyne-11,16-diol	1	1.7	C20 H28 O4	27.457	332.1984	333.2055
6-Deoxyerythronolide B	-	-	C21 H38 O6	27.87	386.267	409.2563
(ent-16betaOH)-16,17-Dihydroxy-9(11)-kauren-19-oic acid	7	-	C20 H30 O4	28.046	334.2141	335.2214
6-Hydroxyenterodiol	19	19.37	C18 H22 O5	28.271	318.1477	341.1367
Cypendazole	-	-	C16 H19 N5 O3	28.371	329.1489	347.1842
12(S)-HpEPE	-	-	C20 H30 O4	28.471	334.2134	335.2206
3,3',4,4'-Tetrahydroxy-5,5'-diisopropyl-2,2'-dimethylbiphenyl	20	20.7	C20 H26 O4	29.248	330.1827	331.19
9-hydroxy-13-oxo-10-octadecenoic acid	1	1.2	C18 H32 O4	29.498	312.2309	335.2201
4,6'-Epoxyorotiniflavan-4-ol	17	17.1	C26 H28 O6	29.599	436.1878	437.1951
2,2-Dimethyl-3,4-bis(4-methoxyphenyl)-2H-1-benzopyran-7-ol acetate	-	-	C27 H26 O5	29.699	430.1791	453.1687
6-HpOME(7E)	-	-	C18 H34 O4	29.899	314.2469	337.2362
Glycyl-Glutamate	4	-	C7 H12 N2 O5	30.476	204.0753	227.0648
Picrasin F	-	-	C22 H30 O8	30.551	422.1935	423.2008
Alfuzosin	18	18.1	C19 H27 N5 O4	30.601	389.2073	407.2417
(ent-16betaOH)-16,17-Dihydroxy9(11)-kauren-19-oicacid	19	19.13	C20 H30 O4	30.726	334.2143	335.2214
**Compound**	**Class**	**Subclass**	**Molecular Formula**	**Retention Time (min)**	**Mass**	**Product Ions (m/z)**
N-(14-Methylhexadecanoyl) pyrrolidine	6	6.3	C21 H41 N O	31.002	323.3203	341.3541
Oryzalic acid B	4	-	C20 H30 O5	31.077	350.2087	351.2162
7-Hydroxyenterolactone	19	19.38	C18 H18 O5	31.703	314.1165	337.1057
ox-LGD2	-	-	C20 H30 O6	31.854	366.2033	367.2108
Ser Val Gln	4	-	C13 H24 N4 O6	32.242	332.1684	333.1755

3-methoxy Prostaglandin F1α	1	1.5	C21 H38 O6	32.468	386.2678	409.2572
15-methyl-15(S)-PGE1	-	-	C21 H36 O5	32.505	368.2567	391.2464
Cryptomeridiol 11-rhamnoside	2	2.5	C21 H38 O6	32.705	386.2668	409.2567
Gentamicin	8	8.3	C21 H43 N5 O7	33.32	477.3161	478.3235
5beta-Chola-3,11-dien-24-oic Acid	-	-	C24 H36 O2	33.407	356.2725	357.2798
[7]-Paradol	21	21.1	C18 H28 O3	33.42	292.2038	315.1944
8-iso Prostaglandin E2 isopropyl ester	6	6.1	C23 H38 O5	33.608	394.2704	395.2778
Thr Glu Leu	4	-	C15 H27 N3 O7	33.808	361.1846	362.1918
2-Methyl-1-phenyl-2-propanyl butyrate	20	20.3	C14 H20 O2	33.959	220.147	243.1362
Sterol 3-beta-D-glucoside	2	2.6	C23 H38 O6	33.984	410.2658	411.2731
Asp Gly Lys	4	-	C12 H22 N4 O6	34.222	318.1528	341.1419
Gln Val Ala	4	-	C13 H24 N4 O5	34.359	316.1736	317.1807
Ser Lys Thr	4	-	C13 H26 N4 O6	34.384	334.184	335.1913
PGF2α-11-acetate methyl ester	6	6.5	C23 H38 O6	34.46	410.2669	411.2743
7-Methylrosmanol	2	2.3	C21 H28 O5	34.485	360.1939	361.2012
11β-Prostaglandin F1β	1	1.5	C20 H36 O5	34.56	356.2557	357.263
8-HpODE	-	-	C18 H32 O4	34.56	312.2288	313.2361
Dihydroisoalantolactone	2	2.3	C15 H22 O2	34.71	234.163	235.1703
Phe Tyr Lys	4	-	C24 H32 N4 O5	34.785	456.2363	457.2437
1-Naphthylacetylspermine	-	-	C22 H34 N4 O	34.91	370.2725	393.2618
Acetylenic acids; 17-Octadecen-9-ynoic acid	-	-	C18 H30 O2	34.936	278.2258	279.2332
3,4-Dimethyl-5-pentyl-2-furanheptanoic acid	1	1.4	C18 H30 O3	35.199	294.2207	295.228
Samandenone	-	-	C22 H33 N O2	35.587	343.2505	344.2578
Stearidonic Acid	1	1.2	C18 H28 O2	35.687	276.21	277.2173
PA(17:2(9Z,12Z)/0:0)	-	-	C20 H37 O7 P	35.763	420.2273	421.2347
13-OxoODE	1	1.2	C18H30O3	35.788	294.2204	317.2096
3',4'-Methylenedioxy-α-pyrrolidinobutiophenone	-	-	C15 H19 N O3	36.113	261.1361	262.1435
Methabenzthiazuron	-	-	C10 H11 N3 O S	36.565	221.063	239.0965
Istamycin KL1	-	-	C13 H28 N4 O6	36.589	336.1996	337.207
Benzthiazuron	-	-	C9 H9 N3 O S	36.99	207.0474	225.081
Ergine	-	-	C16 H17 N3 O	37.09	267.1366	285.1711
Methyl [8]-Shogaol	20	20.8	C20 H30 O3	37.216	318.2187	319.226
Nonoxynol-9	8	8.1	C33 H60 O10	37.516	616.4197	639.4091
Ethyl 1-(ethylthio) propyl disulfide	19	19.39	C7 H16 S3	37.616	196.0422	197.0493
12R-HODE	-	-	C18 H32 O3	37.78	296.2364	319.2258
Iriomoteolide 1a	-	-	C29 H46 O7	38.093	506.3235	507.3309
3-dehydroecdysone	-	-	C27 H42 O6	38.181	462.2973	463.3048
10-F2-dihomo-IsoP	-	-	C23 H40 O5	38.205	396.2889	419.2782
(25S)-5alpha-cholestan-3beta,4beta,6alpha,8beta,15alpha,16beta,26-heptol	-	-	C27 H48 O7	38.318	484.3401	507.3296
Lucidenic acid M	17	17.4	C27 H42 O6	38.456	462.2981	463.3056
Cavipetin D	2	2.2	C25 H38 O5	38.481	418.2718	419.2792
Androst-4-ene-3alpha,17beta-diol diacetate	-	-	C23 H34 O4	38.506	374.2455	375.2529
Deoxycorticosterone	3	3.2	C21 H30 O3	38.644	330.2189	331.2263
(22E,24R)-Stigmasta-4,22-diene-3,6-dione	3	3.4	C29 H44 O2	39.396	424.3352	425.342
**Compound**	**Class**	**Subclass**	**Molecular Formula**	**Retention Time (min)**	**Mass**	**Product Ions (m/z)**
2-(Methoxycarbonyl)-5-methyl-2,4-bis(3-methyl-2-butenyl)-6-(2-methyl-1-oxopropyl)-5-(4-methyl-3-pentenyl) cyclohexanone	2	2.1	C29 H46 O4	39.521	458.3392	481.3285
CB-13	-	-	C26 H24 O2	39.521	368.1793	391.1682

23-demethylgorgosterol	-	-	C29 H48 O	39.596	412.3711	413.3786
14,15-HxA3 (11S)	-	-	C20 H32 O4	39.671	336.2296	337.237
Glyceryl lactopalmitate	32	32.2	C20 H16 N6 O2 S	39.897	404.1063	422.14
Glyceryl lactooleate	1	1.9	C24 H44 O6	39.922	428.3129	429.3204
(2alpha,3alpha,5alpha,22R,23R)-2,3,22,23-Tetrahydroxy-25-methylergost-24(28)en-6-one	3	3.1	C29 H48 O5	40.022	476.3517	499.3409
8beta-Angeloyloxy-15-hydroxy-1alpha,10R-dimethoxy-3-oxo-11(13)-germacren-12,6alpha-olide	2	2.3	C22 H32 O8	40.122	424.2095	425.2169
Thromboxanoic acid skeleton	-	-	C20 H36 O4	40.135	340.2613	341.2686
17,20-dimethyl Prostaglandin F1α	-	-	C22 H40 O5	40.147	384.2875	385.2948
Docosatetraenoyl Ethanolamide	5	5.1	C24 H41 N O2	40.235	375.3134	376.3208
12(13)-EpOME	1	1.4	C18 H32 O3	40.298	296.2347	297.2421
1α-hydroxy-22-[3-(1-hydroxy-1-methylethyl)phenyl]-23,24,25,26,27-pentanorvitamin D3 / 1α-hydroxy-22-[3-(1-hydroxy-1-methylethyl)phenyl]-23,24,25,26,27-pentanorcholecalciferol	-	-	C31 H44 O3	40.46	464.3281	465.3355
N,N-(2,2-dihydroxy-ethyl) arachidonoyl amine	-	-	C24 H41 N O3	40.674	391.3085	392.3159
Uscharidin	-	-	C29 H38 O9	40.699	530.2521	531.2595
Arg Lys Phe	4	-	C21 H35 N7 O4	40.799	449.2764	450.283
Desglucocheirotoxol	3	3.3	C29 H44 O10	40.824	552.2939	575.2835
Pipericine	1	1.1	C22 H41 N O	40.924	335.32	336.3273
PG(O-18:0/0:0)	-	-	C24 H51 O8 P	41.438	498.333	499.3404
Camelledionol	2	2.6	C29 H44 O3	41.475	440.3307	441.3373
Arachidonoylmorpholine	-	-	C24 H39 N O2	41.576	373.2972	374.3046
3-Epimasticadienolic acid	2	2.6	C30 H48 O3	41.625	456.3615	479.3508
Oleic Acid ethyl ester	-	-	C20 H38 O2	41.776	310.2882	311.2956
1-Oleoyl-2-acetyl-sn-glycerol	-	-	C23 H42 O5	41.813	398.3016	399.309
AT-56	-	-	C25 H27 N5	42.052	397.2271	420.2164
Methyl acetyl ricinoleate	-	-	C21 H38 O4	42.152	354.2758	355.2831
ent-8-deoxy-J2-IsoP	-	-	C20 H28 O3	42.327	316.2031	317.2104
Cetiedil	19	-	C20 H31 N O2 S	42.527	349.2068	367.2407
(ent-2b,4S,9a)-2,4,9-Trihydroxy-10(14)-oplopen-3-one 2-(2-methylbutanoate) 9-(3-methyl-2E-pentenoate)	2	2.5	C26 H40 O6	42.628	448.2828	449.29
3-beta-hydroxy-4-beta-methyl-5-alpha-cholest-7-ene-4-alpha-carbaldehyde	3	3.5	C29 H48 O2	42.979	428.3666	429.3739
Austroinulin	2	2.2	C20 H34 O3	43.104	322.2513	345.2411
5,8-Dihydro-6-(4-methyl-3-pentenyl)-1,2,3,4-tetrathiocin	-	-	C10 H16 S4	43.229	264.0124	282.0463
Oleamide	1	1.1	C18 H35 N O	43.38	281.2727	282.2798
Chabrosterol	-	-	C27 H42 O2	43.405	398.3189	399.3262
Lucidone C	3	3.10	C24 H36 O5	43.505	404.2571	427.2463

**Compound**	**Class**	**Subclass**	**Molecular Formula**	**Retention Time (min)**	**Mass**	**Product Ions (m/z)**
Quercetin 3-(2''-p-hydroxybenzoyl-4''-p-coumarylrhamnoside)	17	17.5	C37 H30 O15	43.831	714.1557	715.1633
Sorbitan palmitate	1	1.9	C22 H42 O6	43.906	402.2985	425.2874
Anandamide (20:l, n-9)	-	-	C22 H43 N O2	44.319	353.3296	376.3198

7-Ketodeoxycholic acid	3	3.1	C24 H38 O5	44.332	406.2714	407.2788
Diltiazem	19	-	C22 H26 N2 O4 S	44.532	414.1613	432.1957
Schleicherastatin 3	3	3.1	C29 H50 O3	44.569	446.3767	469.366
Octadecyl fumarate	1	1.9	C22 H40 O4	45.046	368.2924	369.2998
Glaucasterol	-	-	C27 H42 O	45.208	382.3251	383.3324
Stearoylethanolamide	5	5.1	C20 H41 N O2	45.233	327.3153	328.3226
(24E)-3alpha-Acetoxy-15alpha,22S-dihydroxylanosta-7,9(11),24-trien-26-oic acid	2	2.6	C32 H48 O6	45.234	528.3448	529.3514
Cabergoline	19	19.40	C26 H37 N5 O2	45.334	451.2944	469.3281
Polyporusterone A	3	3.1	C28 H46 O6	45.359	478.3282	479.3357
11α-ethynyl-1α,25-dihydroxyvitamin D3 / 11α-ethynyl-1α,25-dihydroxycholecalciferol	-	-	C29 H44 O3	45.384	440.3279	441.3355
(2xi,3xi)-2,3-Dihydroxy-12,18-ursadien-28-oic acid diacetate	2	2.6	C34 H50 O6	45.484	554.3599	555.3671
DG(22:5(7Z,10Z,13Z,16Z,19Z)/14:0/0:0)	19	19.41	C39 H66 O5	45.509	614.4919	637.4811
Conicasterol B	-	-	C29 H44 O	45.572	408.341	409.3477
Makisterone A	-	-	C28 H46 O7	45.585	494.3236	495.3306
22-Docosanolide	1	1.1	C22 H42 O2	45.71	338.3199	339.3271
Polyporusterone C	3	3.5	C28 H44 O6	45.735	476.3136	477.321
Erinacine P	2	2.2	C27 H40 O8	45.81	492.2737	515.2633
13-beta-D-Glucosyloxydocosanoate	-	-	C28 H54 O8	45.835	518.3827	541.372
Asparagoside A	1	1.4	C33 H54 O8	45.998	578.3808	579.3882
Capsi-amide	6	6.5	C17 H35 N O	46.11	269.2729	270.2802
N-stearoyl valine	6	6.1	C23 H45 N O3	46.186	383.3407	406.33
Tigecycline	13	-	C29 H39 N5 O8	46.236	585.2816	603.3155
Spongipregnoloside B	-	-	C33 H52 O11	46.436	624.3533	647.3428
Cyclopassifloic acid C	19	19.3	C31 H52 O7	46.837	536.3706	537.3779
2-Deoxybrassinolide	3	3.3	C28 H48 O5	46.862	464.3493	465.3567
3alpha,7alpha,12alpha,24-tetrahydroxy-24-methyl-5beta-cholestan-26-oic acid	-	-	C28 H48 O6	46.887	480.3446	481.3521
3alpha,7alpha,12alpha-Trihydroxy-5beta-23E-cholestan-26-oic acid	1	1.6	C26 H42 O5	47.012	434.3023	435.3094
Polyporusterone F	3	3.1	C28 H46 O5	47.138	462.3343	463.3416
3beta,15alpha-Diacetoxylanosta-8,24-dien-26-oic acid	2	2.6	C34 H52 O6	47.238	556.3753	557.3826
Stearamide	19	19.42	C18 H37 N O	47.351	283.2887	284.296
27-Nor-5b-cholestane-3a,7a,12a,24,25-pentol	3	3.1	C26 H46 O5	47.514	438.3346	461.3235
Momordol	1	1.7	C26 H48 O5	47.739	440.3513	458.3838
Vitamin D3 glucosiduronate	3	3.6	C33 H52 O7	47.814	560.3693	561.3765
2-Amino-4-oxo-6-(1',2',3'-trihydroxypropyl)-diquinoid-7,8-dihydroxypterin	25	25.1	C9 H15 N5 O6	48.015	289.1017	336.327

**Compound**	**Class**	**Subclass**	**Molecular Formula**	**Retention Time (min)**	**Mass**	**Product Ions (m/z)**
(17Z)-1α,25-dihydroxy-26,27-dimethyl-17,20,22,22,23,23-hexadehydro-24a,24b-dihomovitamin D3 / (17Z)-1α,25-dihydroxy-26,27-dimethyl-	-	-	C31 H46 O3	48.416	466.3464	465.356

17,20,22,22,23,23-hexadehydro-24a,24b-dihomocholecalciferol						
Theasapogenol A	2	2.6	C30 H50 O6	48.554	506.3599	507.3673
6-Deoxodolichosterone	3	3.1	C28 H48 O4	48.691	448.3545	449.3618
Ascariadole epoxide	-	-	C10 H16 O3	48.741	184.1106	185.1179
Hexacosanedioic acid	-	-	C26 H50 O4	48.829	426.3718	427.3792
12β-Hydroxy-3-oxo-5β-cholan-24-oic Acid	3	3.1	C24 H38 O4	48.879	390.278	413.2673
3α,5β-Dihydroxycholan-24-oic Acid	3	3.1	C24 H40 O4	49.142	392.2924	393.2994
Docosanamide	1	1.1	C22 H45 N O	49.694	339.3512	340.3587
13E-Docosenamide	1	1.1	C22 H43 N O	49.944	337.3361	338.3433

Table S2B. Hexane partition [negative mode]

**Compound**	**Class**	**Subclass**	**Molecular Formula**	**Retention Time (min)**	**Mass**	**Product Ions (m/z)**
Sucrose	8	8.3	C12 H22 O11	2.383	342.1161	341.1089

Pyroglutamic acid	6	6.1	C5 H7 N O3	3.134	129.0426	128.0353
(S)-Mevalonic acid	1	1.4	C6 H12 O4	3.185	148.0734	147.0661
6-hydroxy-2-hexynoic acid	-	-	C6 H8 O3	12.868	128.0474	127.0399
C24 H50 O10	19	19.20	C24 H50 O10	14.71	498.3408	497.3336
2-Hydroxydecanedioic acid	1	1.4	C10 H18 O5	15.612	218.115	217.108
Methyl N-(a-methylbutyryl)glycine	1	1.4	C9 H16 O4	17.266	188.1049	187.0977
2,3-dinor Thromboxane B1	-	-	C18 H32 O6	17.479	344.2195	343.2123
Tuberonic acid	19	19.23	C12 H18 O4	17.617	226.1205	225.1133
Polyethylene, oxidized	4	-	C12 H20 O5	17.654	244.1311	243.1238
2,3-dinor Thromboxane B1	1	1.5	C18 H32 O6	17.716	344.2197	343.2124
Leu His Asn	1	1.4	C16 H26 N6 O5	18.343	382.1959	427.1941
Tetranor-PGF1alpha	19	-	C16 H28 O5	18.418	300.1932	359.207
Traumatic Acid	31	31.1	C12 H20 O4	18.606	228.1359	227.1286
2R-hydroxy-10-undecenoic acid	19	19.20	C11 H20 O3	19.27	200.1408	199.1335
2,3-dinor, 6-keto-PGF1α	1	1.5	C18 H30 O6	19.283	342.2038	341.1965
Benzenemethanol, 2-(2-hydroxypropoxy)-3-methyl-	-	-	C11 H16 O3	19.583	196.1099	241.1081
CAY10574	-	-	C9 H10 N6 O	19.671	218.0916	263.0897
6-Epi-7-isocucurbic acid glucoside	1	1.5	C18 H30 O8	19.721	374.1937	373.1861
2,3-Dinor-11b-PGF2a	-	-	C18 H30 O5	20.322	326.2087	325.2015
1-Cyclohexene-1-acrylic acid, 2,6,6-trimethyl-3-oxo-	1	1.2	C12 H16 O3	20.548	208.1096	207.1024
epi-4'-hydroxyjasmonic acid	1	1.2	C12 H18 O4	20.598	226.1203	225.1131
12-hydroxyjasmonic acid	1	1.2	C12 H18 O4	20.874	226.1204	225.1132
Cibaric acid	2	2.1	C18 H28 O5	20.974	324.1931	323.1858
Lactapiperanol D	2	2.2	C18 H28 O5	21.45	324.1934	323.1861
9,12,13-trihydroxy-10,15-octadecadienoic acid	1	1.4	C18 H32 O5	21.625	328.2245	327.2171
Tetranor-PGD1	1	1.5	C16 H26 O5	21.926	298.1777	357.1914
2,2,4,4-Tetramethyl-6-(1-oxobutyl)-1,3,5-cyclohexanetrione	2	2.1	C14 H20 O4	22.477	252.1358	251.1285
2,3-Dinor-TXB2	1	1.5	C18 H30 O6	22.527	342.2039	341.1966
11,12,13-trihydroxy-9-octadecenoic acid	1	1.4	C18 H34 O5	22.64	330.2404	329.2331
O-Geranylvanillin	2	2.1	C18 H24 O3	22.878	288.1721	287.1649
Tetranor Iloprost	1	1.5	C18 H26 O4	22.928	306.1827	305.1755
estra-1,3.5(10)-triene-3,16beta,17beta-triol	3	3.9	C18 H24 O3	23.229	288.1725	287.1652
4'-Hydroxyfenoprofen	20	20.4	C15 H14 O4	23.529	258.0888	257.0814
Furfural diethyl acetal	8	8.1	C9 H14 O3	23.955	170.0942	169.0869
(R)-8-Acetoxycarvotanacetone	2	2.1	C12 H18 O3	24.031	210.1252	209.118
9-hydroperoxy-12,13-dihydroxy-10-octadecenoic acid	1	1.4	C18 H34 O6	24.181	346.2351	345.2278
Hemigossypol	-	-	C15 H16 O4	24.481	260.1043	259.097
5-Hydroxy-1-(4-hydroxyphenyl)-3-decanone	-	-	C16 H24 O3	26.686	264.1721	263.1648
16b-Hydroxyestradiol	19	19.11	C18 H24 O3	27.037	288.1726	287.1652
9-hydroperoxy-10E,12,15Z-octadecatrienoic acid	1	1.4	C18 H30 O4	27.351	310.2137	309.2065
Latanoprost Lactol	1	1.5	C18 H26 O4	27.388	306.1832	305.1756
Embelin	8	8.2	C17 H26 O4	27.501	294.1829	293.1757
5-O-Methylembelin	8	8.2	C18 H28 O4	28.44	308.1982	307.1908
(±)9-HpODE	1	1.2	C18 H32 O4	28.641	312.2296	311.2222

Compound	Class	Subclass	Molecular Formula	Retention Time (min)	Mass	Product Ions (m/z)
His Lys Pro	4	-	C17 H28 N6 O4	30.57	380.2171	379.2096
(3R)-3-isopropenyl-6-oxoheptanoic acid	-	-	C10 H16 O3	31.209	184.1101	183.1026
Undecanedioic acid	1	1.4	C11 H20 O4	31.309	216.136	215.1287

13(S)-HpOTrE	1	1.2	C18 H30 O4	31.46	310.2139	309.2067
5-Hexyltetrahydro-2-oxo-3-furancarboxylic acid	31	31.1	C11 H18 O4	32.049	214.1204	213.113
2,3-dihydroxy stearic acid	1	1.4	C18 H36 O4	32.149	316.2608	315.2535
(±)12,13-DiHOME	1	1.4	C18 H34 O4	32.299	314.245	313.2377
Notoginsenoside R10	3	3.11	C30 H50 O9	32.863	554.3443	553.3371
(23S,25R)-1α,25-dihydroxyvitamin D3 26,23-lactol / (23S,25R)-1α,25-dihydroxycholecalciferol 26,23-lactol	-	-	C27 H42 O5	32.9	446.3023	445.295
10-epi-gamma-Eudesmol	2	2.5	C15 H26 O	33.076	222.1982	267.1963
alpha-licanic acid	-	-	C18 H28 O3	33.151	292.2039	291.1965
Methoprene acid	-	-	C16 H28 O3	33.401	268.2035	267.1961
Lawsone	29	29.2	C10 H6 O3	33.601	174.0315	173.0243
Lophirone D	32	32.3	C24 H16 O6	33.602	400.094	459.1079
(22S)-1α,22,25-trihydroxy-23,24-tetradehydro-24a-homo-20-epivitamin D3 / (22S)-1α,22,25-trihydroxy-23,24-tetradehydro-24a-homo-20-epicholecalciferol	-	-	C28 H42 O4	33.702	442.308	487.306
13(S)-HODE	1	1.2	C18 H32 O3	34.404	296.2354	295.2281
Palmitoyl-EA	19	19.42	C18 H37 N O2	34.441	299.2818	344.2799
Ambrettolic acid	-	-	C16 H30 O3	34.667	270.2191	269.2119
Porrigenin A	2	2.6	C27 H44 O5	34.704	448.3179	447.3107
3,5-Dimethoxy-8,8-dimethyl-2-phenyl-4H,8H-benzo[1,2-b:3,4-b']dipyran-4-one	-	-	C22 H20 O5	35.142	364.1308	363.1236
9-OxoODE	1	1.2	C18 H30 O3	35.256	294.2196	293.2123
18-acetoxy-1α,25-dihydroxyvitamin D3 / 18-acetoxy-1α,25-dihydroxycholecalciferol	-	-	C29 H46 O5	35.28	474.3339	473.3266
cis-9,10-Epoxystearic acid	1	1.2	C18 H34 O3	35.97	298.2506	297.2434
Homodolicholide	9	9.1	C29 H48 O6	36.107	492.3445	491.3373
10S-HODE	1	1.2	C18 H32 O3	36.32	296.2349	295.2277
13(S)-HOTrE	1	1.2	C18 H30 O3	36.483	294.2193	293.2121
(22R)-1α,22,25-trihydroxy-23,24-tetradehydro-24a,24b-dihomo-20-epivitamin D3 / (22R)-1α,22,25-trihydroxy-23,24-tetradehydro-24a,24b-dihomo-20-epicholecalciferol	-	-	C29 H44 O4	36.608	456.3231	455.3158
Octadecanedioic acid	1	1.4	C18 H34 O4	36.684	314.2452	313.2379
Val His Lys	4	-	C17 H30 N6 O4	36.909	382.2326	381.2254
(22S)-1α,22,25-trihydroxy-26,27-dimethyl-23,23,24,24-tetradehydrovitamin D3 / (22S)-1α,22,25-trihydroxy-26,27-dimethyl-23,23,24,24-tetradehydrocholecalciferol	-	-	C29 H44 O4	37.285	456.3235	455.316
Gemfibrozil	11	-	C15 H22 O3	37.961	250.1567	249.1496
Normammein	24	24.1	C21 H26 O5	38.162	358.1776	357.1704
Palmitoyl glucuronide	1	1.8	C22 H42 O7	38.187	418.2924	463.2908
9(S)-HODE	1	1.2	C18 H32 O3	38.237	296.2347	295.2275
1α-hydroxy-2β-(2-hydroxyethoxy)vitamin D3 / 1α-hydroxy-2β-(2-hydroxyethoxy)cholecalciferol	-	-	C29 H48 O4	38.713	460.3543	459.3472
Tephrowatsin B	-	-	C22 H24 O3	39.264	336.1718	381.1701
**Compound**	**Class**	**Subclass**	**Molecular Formula**	**Retention Time (min)**	**Mass**	**Product Ions (m/z)**
10-keto palmitic acid	1	1.4	C16 H30 O3	39.728	270.2193	269.2121
2-Hydroxyhexadecanoic acid	1	1.4	C16 H32 O3	39.828	272.2347	271.2275
Armillarivin	2	2.5	C23 H28 O5	39.866	384.193	383.1857

3-Ethyltridecan-2-one	-	-	C15 H30 O	40.066	226.2294	225.2222
Methylprednisone	2	2.2	C22 H28 O5	40.166	372.1929	371.1856
(24R)-1α,24,25,26-tetrahydroxyvitamin D2 / (24R)-1α,24,25,26-tetrahydroxyergocalciferol	-	-	C28 H44 O5	40.354	460.3182	459.311
N-palmitoyl GABA	6	6.6	C20 H39 N O3	40.893	341.2925	340.2853
Hecogenin Acetate	17	17.2	C29 H44 O5	41.093	472.3185	471.3107
26,27-diethyl-1α,25-dihydroxy-22-thia-20-epivitamin D3 / 26,27-diethyl-1α,25-dihydroxy-22-thia-20-epicholecalciferol	-	-	C30 H50 O3 S	41.193	490.3501	535.3482
Lisuride	14	14.2	C20 H26 N4 O	41.318	338.2118	397.2259
16-hydroxy hexadecanoic acid	1	1.4	C16 H32 O3	41.507	272.2347	271.2274
Pristanal	2	2.2	C19 H38 O	41.895	282.2923	327.2906
Euglobal IIc	9	9.1	C23 H30 O5	42.033	386.2088	385.2016
2-hydroxy behenic	1	1.4	C22 H44 O3	42.609	356.3283	355.3211
Squamosinin A	1	1.7	C36 H62 O8	43.323	622.4429	621.4355
Docosanedioic acid	1	1.4	C22 H42 O4	43.711	370.3085	369.3013
21-Hydroxy-heneicosanoic acid	1	1.4	C21 H42 O3	43.924	342.3127	341.3055
Ricinoleic Acid methyl ester	1	1.4	C19 H36 O3	44.664	312.2657	311.2584
Dihydro-7-Desacetyldeoxygedunin	-	-	C26 H34 O5	44.964	426.2399	425.2327
28-Homobrassinolide	3	3.3	C29 H50 O6	45.027	494.3596	493.3524
DL-2-hydroxy stearic acid	1	1.4	C18 H36 O3	45.553	300.2661	299.2588
1α,25-dihydroxy-26,27-dimethyl-20,21-didehydro-23-oxavitamin D3 / 1α,25-dihydroxy-26,27-dimethyl-20,21-didehydro-23-oxacholecalciferol	-	-	C28 H46 O4	45.628	446.3392	445.3319
S-Japonin	2	2.5	C19 H28 O3 S	46.28	336.1751	381.1735
1α,25-dihydroxy-2α-(3-hydroxypropoxy)-19-norvitamin D3 / 1α,25-dihydroxy-2α-(3-hydroxypropoxy)-19-norcholecalciferol	-	-	C29 H50 O5	47.132	478.3648	477.3575
Chlorogenin	2	2.6	C28 H48 O4	48.71	448.3543	447.3472
2-hydroxyphytanic acid	2	2.2	C20 H40 O3	49.261	328.2972	327.29

Table S2C. EtOAc partition [positive mode]

Compound	Class	Subclass	Molecular Formula	Retention Time (min)	Mass	Product Ions (m/z)
Choline	5	5.2	C5H14NO	1.941	104.1079	104.1074
α-D-Glucose	8	8.3	C6 H12 O6	2.066	180.0635	203.0527
alpha,beta-Trehalose	8	8.3	C12 H22 O11	2.693	342.1174	365.1068
Niacinamide	19	19.14	C6 H6 N2 O	4.221	122.0482	123.0554
2,3-Butanediol glucoside	8	8.3	C10 H20 O7	4.647	252.1216	275.1108
Anhalonidine	-	-	C12 H17 N O3	7.328	223.1212	224.1286
Butamben	20	20.1	C11 H15 N O2	7.704	193.1107	194.118
2,3-Butanediol glucoside	14	14.6	C10 H20 O7	7.729	252.1212	275.1105
Ethyl (S)-3-hydroxybutyrate glucoside	1	1.8	C12 H22 O8	10.41	294.1318	317.121
PE(18:4(6Z,9Z,12Z,15Z)/0:0)	-	-	C23 H40 N O7 P	13.254	473.2561	496.2445
diethyl 2,6-dimethyl-4-oxo-4h-pyran-3,5-dicarboxylate	-	-	C13 H16 O6	13.466	268.0952	291.0844
Octanoylglucuronide	8	8.3	C14 H24 O8	13.768	320.1479	343.1371
Trp-P-1	23	23.4	C13 H13 N3	13.93	211.1117	212.119
Glucocaffeic acid	8	8.3	C15 H18 O9	13.968	342.0955	365.0846
1,4-Naphthoquinone	29	29.2	C10 H6 O2	14.043	158.0371	159.0443
3-Hydroxy-4-butanolide	8	8.3	C10 H16 O8	14.281	264.0852	287.0744
N1-Caffeoyl-N10-feruloylspermidine	12	12.2	C26 H33 N3 O6	14.444	483.237	484.2443
N1,N10-Diferuloylspermidine	12	12.2	C27 H35 N3 O6	14.82	497.2541	498.2614
alpha-Peroxyachifolide	2	2.3	C20 H24 O7	15.646	376.153	399.1423
1,5-Dibutyl methyl hydroxycitrate	6	6.4	C15 H26 O8	15.847	334.1634	357.1526
Pentyl heptanoate	1	1.9	C12 H24 O2	17.074	200.178	218.2119
2,3-dinor Thromboxane B1	-	-	C18 H32 O6	17.651	344.2202	367.2094
methyl 9,11-epidioxy-12,15-dihydroperoxy-5,7,13-eicosatrienoate	-	-	C21 H34 O8	17.776	414.2257	437.215
Tetranor-PGF1alpha	-	-	C16 H28 O5	18.227	300.194	323.1833
Pterostilbene Glycinate	7	-	C18 H19 N O4	18.377	313.132	336.1212
Norcapillene	-	-	C11 H8	18.578	140.063	141.0703
Citrusinine I	14	14.4	C16 H15 N O5	18.653	301.0956	324.0849
Gln Ala Trp	4	-	C19 H25 N5 O5	18.929	403.1862	421.2196
Acetylcaranine	6	6.1	C18 H19 N O4	19.029	313.1323	336.1215
10-hydroxy-hexadecan-1,16-dioic acid	-	-	C16 H30 O5	19.38	302.2098	325.199
formyl 7-oxo-11E-tetradecenoate	-	-	C15 H26 O3	19.53	254.1888	277.1781
Myxochelin B	-	-	C20 H25 N3 O6	19.718	403.1746	421.2087
Armillane	2	2.2	C23 H32 O7	20.081	420.2134	421.2202
Myristicanol B	-	-	C22 H28 O7	20.382	404.184	427.1732
Samin	19	-	C13 H14 O5	20.633	250.0848	273.0741
Decyl isobutyrate	-	-	C14 H28 O2	20.733	228.2097	246.2435
(9R,10S,12Z)-9,10-Dihydroxy-8-oxo-12-octadecenoic acid	1	1.2	C18 H32 O5	21.134	328.2259	351.2153
Phe Arg Thr	4	-	C19 H30 N6 O5	21.835	422.2287	423.2358
9S,10S,11R-trihydroxy-12Z-octadecenoic acid	1	1.4	C18 H34 O5	22.186	330.2418	353.2312
1-Hexadecylamine	5	5.1	C16 H35 N	22.361	241.2772	242.2845
Luvangetin	-	-	C15 H14 O4	22.8	258.0898	281.079
Dihydrocapsiate	21	21.1	C18 H28 O4	23.388	308.1992	331.1885
C16 Sphinganine	5	5.1	C16 H35 N O2	24.015	273.2676	296.2568
Palmitic amide	1	1.1	C16 H33 N O	24.04	255.2567	256.264
Xestoaminol C	5	5.1	C14 H31 N O	24.253	229.2415	230.2488
Phytosphingosine	5	5.1	C18 H39 N O3	24.29	317.2938	318.3012
7-Ethyl-4-tridecen-6-one	-	-	C15 H28 O	24.566	224.2148	242.2486
2-Aminohexadecanoic acid	1	1.9	C16 H33 N O2	24.892	271.2519	272.2592
Palmitoleamide	1	1.1	C16 H31 N O	24.929	253.2411	254.2483
Limonen-6-ol-pivalate	-	-	C15 H24 O2	25.042	236.1784	259.1676

Compound	Class	Subclass	Molecular Formula	Retention Time (min)	Mass	Product Ions (m/z)
C17 Sphinganine	5	5.1	C17 H37 N O2	25.143	287.2828	288.2901
Prosopinine	-	-	C16 H33 N O3	25.318	287.247	288.2541
19-noraldosterone	3	3.2	C20 H26 O5	25.493	346.1786	369.1679
17-hydroxy-heptadecanoic acid	1	1.4	C17 H34 O3	26.195	286.2515	304.2853
Gingerenone B	19	19.3	C22 H26 O6	26.746	386.1735	409.1628
(R)-benzyl Mandelate	20	20.9	C15 H14 O3	27.798	242.0948	265.084
Calamendiol	2	2.5	C15 H26 O2	28.6	238.1938	261.1828
15-methyl-15(S)-PGE1	-	-	C21 H36 O5	28.914	368.2567	391.246
N-Methyldioctylamine	5	5.1	C17 H37 N	29.402	255.2934	256.3007
2,2-Dimethyl-3,4-bis(4-methoxyphenyl)-2H-1-benzopyran-7-ol acetate	-	-	C27 H26 O5	29.477	430.1785	453.168
Clausarinol	24	24.2	C24 H30 O6	29.515	414.2052	437.1945
15-methyl-15S-PGE2	-	-	C21 H34 O5	30.317	366.2413	389.2303
Alfuzosin	18	18.1	C19 H27 N5 O4	30.404	389.2069	407.2407
Cincassiol B	2	2.2	C20 H32 O8	30.479	400.2106	423.1998
8-HpODE	1	1.2	C18 H32 O4	30.58	312.2309	335.22
1-Naphthylacetylspermine	6	6.5	C22 H34 N4 O	31.031	370.2721	393.2614
Leu Ala Arg	4	-	C15 H30 N6 O4	31.219	358.2337	359.2409
Stearidonic Acid	1	1.2	C18 H28 O2	32.008	276.2096	277.2168
Kolanone	-	-	C33 H42 O4	32.045	502.3059	520.3399
13,16-Octadecadiynoic acid	1	1.4	C18 H28 O2	32.408	276.2097	277.2169
LysoPE(0:0/16:0)	27	27.1	C21 H44 N O7 P	32.509	453.2865	454.2939
Glu His Glu	4	-	C16 H23 N5 O8	32.559	413.154	431.1877
9-Nonadecene	19	19.36	C19 H38	32.76	266.2984	284.3322
Gentamicin	8	8.3	C21 H43 N5 O7	33.01	477.3149	478.3223
[7]-Paradol	21	21.1	C18 H28 O3	33.236	292.2044	315.1935
18:1(5Z)(9Me,13Me,17Me)	-	-	C21 H40 O2	33.636	324.3033	342.3372
(Z)-2-Tetracos-15-enamidoethanesulfonic acid	-	-	C26 H51 N O4 S	33.887	473.3523	496.3416
PE(19:0/0:0)	-	-	C24 H50 N O7 P	34	495.3338	518.3231
1-Palmitoyllysophosphatidylcholine	27	27.2	C24H51NO7P	34.125	496.3411	496.3405
Acetylenic acids; 17-Octadecen-9-ynoic acid	-	-	C18 H30 O2	34.238	278.2251	279.2324
9-Docosene	19	19.7	C22 H44	34.363	308.3455	326.3794
Citrubuntin	24	-	C15 H14 O3	34.914	242.0946	243.1018
Polysorbate 60	1	1.9	C22 H42 O8	34.965	434.2885	457.2778
1-Naphthylacetylspermine	1	1.2	C22 H34 N4 O	35.114	370.2719	393.2611
13-OxoODE	-	-	C18 H30 O3	35.39	294.22	317.2093
1a,1b-dihomo-PGE1	6	6.3	C22 H38 O5	35.415	382.2725	405.2616
Dilauryl 3,3'-thiodipropionate	3	3.12	C30 H58 O4 S2	35.515	546.3752	547.3822
Ginsenoside I	3	3.12	C48 H82 O20	35.716	978.5404	507.3039
PI(O-20:0/22:6 (4Z,7Z,10Z,13Z,16Z,19Z))	-	-	C51 H89 O12 P	35.891	924.6068	485.2926
Citronellyl cinnamate	1	1.3	C19 H26 O2	36.016	286.1938	309.1832
PtdIns-(5)-P1 (1,2-dipalmitoyl)	-	-	C41 H80 O16 P2	36.029	890.489	463.2784
Diospolysaponin A	-	-	C40 H66 O16	36.418	802.4366	419.2521
Pseudoginsenoside RT3	2	2.6	C41 H70 O13	36.631	770.4793	771.4867
Dihydroartemisinin	2	2.5	C15 H24 O5	36.918	284.1625	285.1705
Cyclopassifloside II	2	2.4	C37 H62 O11	37.082	682.4276	683.4351
Diisobutyl phthalate	20	20.1	C16 H22 O4	37.257	278.1524	301.1416
Nonoxynol-9	8	8.1	C33 H60 O10	37.27	616.4196	639.409
(25S)-5alpha-cholestan-3beta,4beta,6alpha,8beta,15alpha,16beta,26-heptol	-	-	C27 H48 O7	37.796	484.341	507.3303
Leu Trp Lys	4	-	C23 H35 N5 O4	38.084	445.2704	463.3042

Compound	Class	Subclass	Molecular Formula	Retention Time (min)	Mass	Product Ions (m/z)
10-F2-dihomo-IsoP	-	-	C23 H40 O5	38.247	396.2882	419.2775
Montanol	1	1.4	C21 H36 O4	38.385	352.2621	375.2514
Annosquamosin B	2	2.2	C19 H32 O3	38.447	308.2357	331.225
(3b,6b,8a,12a)-8,12-Epoxy-7(11)-eremophilene-6,8,12-trimethoxy-3-ol	-	-	C18 H30 O5	38.522	326.2099	349.1992
Polidocanol	8	8.1	C30 H62 O10	38.936	582.4344	605.4237
8beta-Angeloyloxy-15-hydroxy-1alpha,10R-dimethoxy-3-oxo-11(13)-germacren-12,6alpha-olide	2	2.3	C22 H32 O8	39.625	424.2094	447.1994
Glyceryl lactooleate	1	1.9	C24 H44 O6	39.825	428.3123	429.3197
Val Val Trp	4	-	C21 H30 N4 O4	39.938	402.2268	425.2161
17,20-dimethyl Prostaglandin F1α	1	1.5	C22 H40 O5	39.963	384.2863	385.2936
Thromboxanoic acid skeleton	-	-	C20 H36 O4	40.051	340.26	341.2673
Anandamide (20:l, n-9)	5	5.1	C22 H43 N O2	40.151	353.3302	376.3194
Lys Lys Ser	4	-	C15 H31 N5 O5	40.364	361.2308	379.2646
N-(2-Methoxyphenyl)-N'-(2-naphtyl)urea	-	-	C18 H16 N2 O2	40.401	292.1221	293.1291
Aplidiasphingosine	-	-	C22 H43 N O3	40.564	369.325	392.3141
Erinacine G	8	8.3	C25 H36 O8	40.614	464.2422	487.2318
Aplidiasphingosine	4	-	C22 H43 N O3	40.802	369.3251	392.3144
Ile Phe Trp	-	-	C26 H32 N4 O4	40.827	464.2426	487.2321
N-tert-Butyloxycarbonyl-deacetyl-leupeptin	-	-	C23 H44 N6 O5	41.028	484.3363	265.1573
PE(21:0/0:0)	-	-	C26 H54 N O7 P	41.328	523.3642	524.3715
Desglucocheirotoxol	3	3.3	C29 H44 O10	41.328	552.2948	575.2845
Anandamide (20:2, n-6)	5	5.1	C22 H41 N O2	41.429	351.3147	374.304
1-Oleoyl-2-acetyl-sn-glycerol	-	-	C23 H42 O5	41.905	398.3017	399.3091
Lys Val Trp	4	-	C22 H33 N5 O4	42.506	431.2545	449.2882
9-Pentacosene	19	19.36	C25 H50	42.531	350.3919	368.4258
Falcarindiol	1	1.7	C17 H24 O2	42.669	260.1783	261.1856
Terbucarb	-	-	C17 H27 N O2	42.932	277.2047	278.2117
Purpureacin 2	1	1.7	C37 H66 O8	42.932	638.4758	661.4651
Oleamide	1	1.1	C18 H35 N O	43.333	281.2725	282.2798
Octadecyl fumarate	1	1.9	C22 H40 O4	44.962	368.291	369.2984
(13R,14R)-7-Labdene-13,14,15-triol	2	2.2	C20 H36 O3	45.037	324.2648	325.2722
Met Arg Val	4	-	C16 H32 N6 O4 S	45.112	404.2213	427.2104
Polyporusterone A	3	3.1	C28 H46 O6	45.438	478.3276	479.3349
Glutathionylaminopropylcadaverine	-	-	C18 H36 N6 O5 S	45.613	448.2481	471.2372
22-Docosanolide	1	1.1	C22 H42 O2	45.638	338.3192	339.3265
13-beta-D-Glucosyloxydocosanoate	-	-	C28 H54 O8	45.738	518.3825	541.3717
N-stearoyl valine	6	6.1	C23 H45 N O3	46.026	383.3407	406.3299
13E-Docosenamide	1	1.1	C22 H43 N O	46.139	337.3351	338.3424
Spongipregnoloside B	-	-	C33 H52 O11	46.54	624.3522	647.3418
Stearamide	19	19.42	C18 H37 N O	46.716	283.2885	284.2958
Momordol	1	1.7	C26 H48 O5	47.041	440.3506	463.3397
Pipericine	1	1.1	C22 H41 N O	47.718	335.3196	336.3268
Polyporusterone F	3	3.1	C28 H46 O5	48.131	462.3335	463.3403
Theasapogenol A	2	2.6	C30 H50 O6	48.544	506.3587	507.3659
TG(8:0/8:0/8:0)	19	19.43	C27 H50 O6	48.62	470.361	493.3504
12β-Hydroxy-3-oxo-5β-cholan-24-oic Acid	-	-	C24 H38 O4	48.758	390.2777	413.267
Dioctyl hexanedioate	1	1.3	C22 H42 O4	49.021	370.3089	393.298
Eicosapentaenoyl Serotonin	23	23.5	C30 H40 N2 O2	49.584	460.3095	461.317

Table S2D. EtOAc partition [negative mode]

Compound	Class	Subclass	Molecular Formula	Retention Time (min)	Mass	Product Ions (m/z)
Sucrose	8	8.3	C12 H22 O11	2.72	342.1161	387.1143
Citbismine C	14	14.4	C37 H36 N2 O11	2.745	684.232	683.2249
Pyroglutamic acid	6	6.1	C5 H7 N O3	4.399	129.0426	128.0353
Succinic acid	6	6.3	C4 H6 O4	4.499	118.0264	117.0192
Erythrityl Tetranitrate	19	19.44	C4 H6 N4 O12	4.549	301.9995	300.9919
Adenine	26	26.1	C5 H5 N5	4.6	135.0543	134.047
Pyrocatechol	21	21.6	C6 H6 O2	10.562	110.0366	109.0294
Fenpyroximate	8	8.1	C24 H27 N3 O4	11.227	421.1999	480.2137
Cadabicine	8	8.1	C25 H29 N3 O4	11.765	435.2156	480.2138
3,4-Dihydroxybenzaldehyde	8	8.2	C7 H6 O3	12.116	138.0316	137.0242
Esculetin	24	24.1	C9 H6 O4	13.081	178.0266	177.0193
Benexate	-	-	C23 H27 N3 O4	13.119	409.2002	468.214
4-Aminocatechol	-	-	C6 H7 N O2	13.268	125.0476	124.0403
o-Vinylanisole	11	11.1	C9 H10 O	13.883	134.0735	133.0662
2-Deoxy-D-glucose 6-phosphate	-	-	C6 H13 O8 P	13.92	244.0347	243.0275
Lunarine	-	-	C25 H31 N3 O4	13.92	437.2322	482.2306
N1,N10-Dicoumaroylspermidine	12	12.2	C25 H31 N3 O4	14.221	437.2323	482.2307
Citrusin B	19	-	C27 H36 O13	14.847	568.2161	567.209
Methylpicraquassioside A	8	8.3	C19 H24 O10	15.424	412.1368	411.1296
Dihydrojasmonic Acid, Methyl Ester	1	1.2	C13 H22 O3	15.624	226.1572	271.1554
Cassitoroside	8	8.3	C25 H32 O14	16.15	556.1792	555.1721
L-Citronellol glucoside	2	2.4	C16 H30 O6	16.351	318.2043	317.1971
Marchantin A	-	-	C28 H24 O5	16.426	440.1617	439.1548
xi-Linalool 3-[rhamnosyl-(1->6)-glucoside]	1	1.8	C22 H38 O10	16.776	462.2473	461.2395
Neryl rhamnosyl-glucoside	2	2.4	C22 H38 O10	17.152	462.247	461.2393
Methyl N-(a-methylbutyryl)glycine	1	1.4	C9 H16 O4	17.228	188.1051	187.0979
2,3-dinor Thromboxane B1	4	-	C18 H32 O6	17.453	344.2202	343.2129
His Asn Val	29	-	C15 H24 N6 O5	18.155	368.1811	367.1738
2-Methylnaphthalene	2	2.5	C11 H10	18.179	142.0777	141.0709
Lubiminol	32	32.2	C15 H26 O3	18.192	254.1887	299.1869
Sulfaphenazole	-	-	C15 H14 N4 O2 S	18.355	314.0829	313.0755
3-(1,1-Dimethylallyl)scopoletin 7-glucoside	24	24.3	C21 H26 O9	18.455	422.1578	421.1505
Ismine	-	-	C15 H15 N O3	18.58	257.1056	256.0983
Citpressine I	14	14.4	C16 H15 N O5	18.581	301.0957	300.0885
2-glyceryl-6-keto-PGF1α	1	1.5	C23 H40 O8	18.906	444.2726	489.2709
N-Feruloyltyramine	12	12.2	C18 H19 N O4	19.032	313.1315	312.1243
Citpressine II	14	14.4	C17 H17 N O5	19.057	315.111	314.1038
9-hydroxy-hexadecan-1,16-dioic acid	-	-	C16 H30 O5	19.332	302.2096	301.2024
2-Butanone, 4-(6-hydroxy-2-naphthalenyl)-	-	-	C14 H14 O2	19.345	214.0994	213.0921
Sebacic acid	1	1.4	C10 H18 O4	19.433	202.1205	201.1132
Xylocarpus A	-	-	C31 H38 O11	19.696	586.2416	645.2556
2,3,4-Trihydroxy-4'-Ethoxybenzophenone	-	-	C15 H14 O5	19.796	274.0847	273.0775
Cinnzeylanol	2	2.2	C20 H32 O7	20.184	384.2159	443.229
Hesperetin	17	17.4	C16 H14 O6	20.359	302.0789	301.0716
Baccatin III	2	2.2	C31 H38 O11	20.435	586.2415	645.2555
7-Methyl-2-benzofurancarboxaldehyde	19	-	C10 H8 O2	20.66	160.0524	159.0452
Deacetoxy-7-Oxogedunin	-	-	C26 H30 O6	20.736	438.2042	437.1969
beta-Lapachone	19	19.45	C15 H14 O3	20.936	242.0943	241.0871

Compound	Class	Subclass	Molecular Formula	Retention Time (min)	Mass	Product Ions (m/z)
9,12,13-trihydroxy-10,15-octadecadienoic acid	1	1.2	C18 H32 O5	21.136	328.2258	327.2185
His Ile Gln	4	-	C17 H28 N6 O5	21.161	396.2127	395.2055
Oleandolide	-	-	C20 H34 O7	21.738	386.2308	445.2445
11,12,13-trihydroxy-9-octadecenoic acid	1	1.2	C18 H34 O5	22.239	330.2412	329.234
alpha-Ionol O-[arabinosyl-(1->6)-glucoside]	1	1.8	C24 H40 O10	23.09	488.2617	487.2544
4'-Hydroxyfenoprofen	20	20.4	C15 H14 O4	23.216	258.0893	257.0821
Ambrettolic acid	-	-	C16 H30 O3	24.493	270.2193	329.2332
Isoliquiritigenin	30	30.1	C15 H12 O4	24.894	256.0734	255.0661
Sarcostin	6	6.1	C21 H34 O6	25.195	382.2353	427.2335
5-O-Methylembelin	2	2.7	C18 H28 O4	25.321	308.1988	307.1912
Embelin	2	2.7	C17 H26 O4	27.25	294.1832	293.176
(±)9-HpODE	1	1.2	C18 H32 O4	27.801	312.2301	311.2227
9-hydroperoxy-10E,12,15Z-octadecatrienoic acid	-	-	C18 H30 O4	28.102	310.2143	309.2071
Gingerglycolipid A	19	19.35	C33 H56 O14	28.353	676.3672	721.3655
Buthiobate	27	27.1	C21 H28 N2 S2	28.803	372.1701	431.1837
PE(18:3(9Z,12Z,15Z)/0:0)	27	27.1	C23 H42 N O7 P	29.004	475.2704	474.2633
Tyr Cys Glu	4	-	C17 H23 N3 O7 S	31.309	413.1266	458.1246
Lapachol	29	29.2	C15 H14 O3	31.71	242.0947	241.0875
Mupirocin	1	1.4	C26 H44 O9	31.785	500.2989	559.3128
LysoPE(0:0/16:0)	27	27.1	C21 H44 N O7 P	31.81	453.2857	452.2784
9(S)-HOTrE	1	1.2	C18 H30 O3	32.011	294.2202	293.213
PE(18:2(9Z,12Z)/0:0)	27	27.1	C23 H44 N O7 P	32.211	477.2857	476.2785
Strophanthidin Semicarbazide	3	3.3	C24 H35 N3 O6	32.487	461.2519	520.2658
alpha-licanic acid	-	-	C18 H28 O3	33.012	292.2042	291.1968
Deoxygomisin A	-	-	C23 H28 O6	33.088	400.1886	399.1814
alpha,alpha'-Trehalose 6-palmitate	-	-	C28 H52 O12	33.389	580.3458	625.3441
9-OxoOTrE	-	-	C18 H28 O3	33.639	292.204	291.1967
13(S)-HODE	1	1.2	C18 H32 O3	34.216	296.2359	295.2287
PA(18:3(9Z,12Z,15Z)/0:0)	27	27.3	C21 H37 O7 P	34.754	432.2278	431.2203
3,5-Dimethoxy-8,8-dimethyl-2-phenyl-4H,8H-benzo[1,2-b:3,4-b']dipyran-4-one	-	-	C22 H20 O5	34.917	364.1316	363.1244
Mangostinone	9	9.1	C23 H24 O5	35.405	380.1633	379.1563
Notoginsenoside T1	2	2.6	C36 H60 O10	35.543	652.419	651.4119
(E)-4-(3,7-Dimethyl-2,6-octadienyl)-1,3,5-trihydroxyxanthone	9	9.1	C23 H24 O5	35.718	380.1632	379.1562
8-Desoxygartanin	9	9.1	C23 H24 O5	35.919	380.163	379.156
Praecanson A	-	-	C23 H24 O5	36.408	380.1636	379.1563
7-Methoxypraecansone B	-	-	C23 H24 O5	36.596	380.1632	379.1562
1,1-Diphenyl-2-(4-methoxyphenyl)propene	-	-	C22 H20 O	37.422	300.1513	345.1496
PE(18:0/0:0)	-	-	C23 H48 N O7 P	37.448	481.3168	480.3097
Desacetylvindoline	-	-	C23 H30 N2 O5	37.761	414.217	413.2098
Tibolone	3	3.10	C21 H28 O2	38.024	312.209	311.2017
PG(16:0/0:0)	-	-	C22 H45 O9 P	38.224	484.2802	483.273
1,3,8-Trihydroxy-4-methyl-2,7-diprenylxanthone	9	9.1	C24 H26 O5	38.324	394.1787	393.1717
(25S)-5alpha-cholestan-3beta,4beta,6alpha,7beta,8beta,15alpha,16beta,26-octol	-	-	C27 H48 O8	38.625	500.3355	559.3495
Kukoamine D	21	21.6	C28 H42 N4 O6	39.803	530.3122	529.3052

Compound	Class	Subclass	Molecular Formula	Retention Time (min)	Mass	Product Ions (m/z)
Kushenol A	-	-	C25 H28 O5	40.667	408.1938	407.1865
PA(18:2(9Z,12Z)/0:0)	27	27.3	C21 H39 O7 P	40.83	434.2435	433.2358
3-O-(2-O-(2E-decenoyl)-alpha-L-rhamnopyranosyl)-3-hydroxydecanoic acid	-	-	C36 H64 O10	41.03	656.4491	655.4418
Pinolenic Acid	1	1.2	C18 H30 O2	41.206	278.2247	277.2174
2-Prenyl-6a-hydroxyphaseollidin	28	28.1	C25 H28 O5	41.331	408.1946	407.1871
16-hydroxy hexadecanoic acid	1	1.4	C16 H32 O3	41.356	272.2353	271.2281
3R-hydroxy-eicosanoic acid	1	1.4	C20 H40 O3	41.77	328.2979	327.2908
3'-Geranyl-3,4,2',4'-tetrahydroxychalcone	30	30.1	C25 H28 O5	42.509	408.1944	407.1873
Squamosinin A	1	1.7	C36 H62 O8	42.86	622.4438	621.4365
Met Thr Arg	4	-	C15 H30 N6 O5 S	43.01	406.199	405.1928
Cedrediprenone	-	-	C25 H28 O5	43.323	408.1941	407.1871
Docosanedioic acid	1	1.4	C22 H42 O4	43.511	370.3088	369.3016
Isohyodeoxycholic acid	3	3.1	C24H40O4	43.536	392.2904	391.2832
1α-hydroxy-23-[3-(1-hydroxy-1-methylethyl)phenyl]-22,22,23,23-tetradehydro-24,25,26,27-tetranorvitamin D3 / 1α-hydroxy-23-[3-(1-hydroxy-1-methylethyl)phenyl]-22,22,23,23-tetradehydro-24,25,26,27-tetranorcholecalciferol	-	-	C32 H42 O3	44.513	474.3138	473.3067
Ricinoleic acid methyl ester	-	-	C19 H36 O3	44.576	312.2664	311.2591
DL-2-hydroxy stearic acid	1	1.4	C18 H36 O3	45.428	300.2666	299.2594
2-hydroxy behenic	1	1.4	C22 H44 O3	45.641	356.3296	355.3224
Lithocholic acid sulfate	3	3.1	C24 H40 O6 S	45.678	456.2544	455.2473
Tetracosanedioic acid	1	1.4	C24 H46 O4	46.844	398.3396	397.3325
2-hydroxy-nonadecanoic acid	-	-	C19 H38 O3	47.27	314.2817	313.2744
2-hydroxy-tricosanoic acid	-	-	C23 H46 O3	47.445	370.3446	369.3373
15-methoxy-tricosanoic acid	-	-	C24 H48 O3	49.55	384.3602	383.353

Table S3. Class and subclass of metabolites found *in R. nasutus* leaf and SCC extracts by UHPLC-QToF-MS analysis

No	Class	Subclass
**1**	Class fatty acyls	1.1 Subclass fatty amides
		1.2 Subclass lineolic acids and derivatives
		1.3 Subclass fatty alcohol esters
		1.4 Subclass fatty acids and conjugates
		1.5 Subclass eicosanoids
		1.6 Subclass fatty acyl thioesters
		1.7 Subclass fatty alcohol
		1.8 Subclass fatty acyl glycosides
		1.9 Subclass fatty acid esters
**2**	Class prenol lipids	2.1 Subclass monoterpenoids
		2.2 Subclass diterpenoids
		2.3 Subclass terpene lactones
		2.4 Subclass terpene glycosides
		2.5 Subclass sesquiterpenoids
		2.6 Subclass triterpenoids
		2.7 Subclass quinone and hydroquinone lipids
		2.8 Subclass sesterterpenoids
		2.9 Subclass polyprenols
		2.10 Subclass tetraterpenoids
**3**	Class steroids and steroid derivatives	3.1 Subclass bile acids, alcohols and derivatives
		3.2 Subclass hydroxysteroids
		3.3 Subclass steroid lactones
		3.4 Subclass stigmastanes and derivatives

		3.5 Subclass cholestane steroids<br>3.6 Subclass vitamin D and derivatives<br>3.7 Subclass androstane steroids<br>3.8 Subclass sulfated steroids<br>3.9 Subclass estrane steroid<br>3.10 Subclass oxosteroids<br>3.11 Subclass steroidal glycosides<br>3.12 Subclass steroidal alkaloids
**4**	Class peptide	-
**5**	Class organonitrogen compounds	5.1 Subclass amines<br>5.2 Subclass quaternary ammonium salts
**6**	Class carboxylic acids and derivatives	6.1 Subclass amino acids, peptides, and analogues<br>6.2 Subclass carboxylic acids<br>6.3 Subclass dicarboxylic acids and derivatives<br>6.4 Subclass tricarboxylic acids and derivatives<br>6.5 Subclass carboxylic acid derivatives<br>6.6 Subclass gamma amino acids and derivatives
**7**	Class stilbenes	7.1 Subclass stilbene glycosides
**8**	Class organooxygen compounds	8.1 Subclass ethers<br>8.2 Subclass carbonyl compounds<br>8.3 Subclass carbohydrates and carbohydrate conjugates
**9**	Class benzopyrans	9.1 Subclass 1-benzopyrans<br>9.2 Subclass 2-benzopyrans
**10**	Class tetrapyrroles and derivatives	10.1 Subclass porphyrins

		10.2 Subclass chlorins
**11**	Class phenol ethers	11.1 Subclass anisoles
**12**	Class cinnamic acids and derivatives	12.1 Subclass cinnamic acid esters
		12.2 Subclass hydroxycinnamic acids and derivatives
		12.3 Subclass cinnamic acids
**13**	Class tetracyclines	-
**14**	Class quinolines and derivatives	14.1 Subclass quinoline carboxylic acids
		14.2 Subclass indoloquinolines
		14.3 Subclass hydroxyquinolines
		14.4 Subclass benzoquinolines
		14.5 Subclass quinolines and derivatives
		14.6 Subclass quinolones and derivatives
**15**	Class phenanthrenes and derivatives	15.1 Subclass hydrophenanthrenes
**16**	Class macrolides and analogues	-
**17**	Class flavonoids	17.1 Subclass flavans
		17.2 Subclass flavonoid glycosides
		17.3 Subclass pyranoflavonoid
		17.4 Subclass o-methylated flavonoids
		17.5 Subclass flavones
**18**	Class diazanaphthalenes	18.1 Subclass benzodiazines
**19**	Others	19.1 Subclass acetophenones
		19.2 Subclass indazoles
		19.3 Subclass linear diarylheptanoids
		19.4 Subclass diphenylmethanes
		19.5 Subclass biphenols

19.6 Subclass pyranoisoflavonoids

19.7 Subclass unsaturated aliphatic hydrocarbons

19.8 Subclass phosphate esters

19.9 Subclass glycerophosphates

19.10 Subclass delta valerolactones

19.11 Subclass lipids and lipid-like molecules

19.12 Subclass diradylglycerols

19.13 Subclass N-acylpyrrolidines

19.14 Subclass pyridinecarboxylic acids and derivatives

19.15 Subclass oxazoles

19.16 Subclass chalcones and dihydrochalcones

19.17 Subclass anthraquinones

19.18 Subclass gamma-keto acids and derivatives

19.19 Subclass phenols

19.20 Subclass medium-chain hydroxy acids and derivatives

19.21 Subclass naphthoquinones

19.22 Subclass pyridinecarboxylic acids and derivatives

19.23 Subclass medium-chain keto acids and derivatives

19.24 Subclass non-metal nitrites

19.25 Subclass pyridinecarboxylic acids and derivatives

19.26 Subclass oxazolines

19.27 Subclass thiophosphoric acid esters

19.28 Subclass benzo-1,4-dioxanes

19.29 Subclass isoindolines

		19.30 Subclass phenylpyrrolidines<br>19.31 Subclass imidazolidines<br>19.32 Subclass hybrid peptides<br>19.33 Subclass gamma butyrolactones<br>19.34 Subclass dibenzylbutanediol lignans<br>19.35 Subclass glycosylglycerols<br>19.36 Subclass alkanes<br>19.37 Subclass dibenzylbutane lignans<br>19.38 Subclass tetrahydrofuran lignans<br>19.39 Subclass dialkyldisulfides<br>19.40 Subclass lysergic acids and derivatives<br>19.41 Subclass diradylglycerol<br>19.42 Subclass carboximidic acids<br>19.43 Subclass triradylcglycerols<br>19.44 Subclass organic nitrates<br>19.45 Subclass naphthopyranones
**20**	Class benzene and substituted derivatives	20.1 Subclass benzoic acids and derivatives<br>20.2 Subclass benzoyl derivatives<br>20.3 Subclass phenylpropanes<br>20.4 Subclass diphenylethers<br>20.5 Subclass benzenesulfonamides<br>20.6 Subclass acetophenones<br>20.7 Subclass biphenols<br>20.8 Subclass methoxybenzenes<br>20.9 Subclass benzyloxycarbonyls

**21**	Class phenols	21.1 Subclass methoxyphenols<br>21.2 Subclass nitrophenols<br>21.3 Subclass benzenetriols and derivatives<br>21.4 Subclass 1-hydroxy-4-unsubstituted benzenoids<br>21.5 Subclass tyrosols and derivatives<br>21.6 Subclass benzenediols
**22**	Class phenol ester	-
**23**	Class indoles and derivatives	23.1 Subclass indole carboxylic acids and derivatives<br>23.2 Subclass indolyl carboxylic acids and derivatives<br>23.3 Subclass hydroxyindoles<br>23.4 Subclass pyridoindoles<br>23.5 Subclass indoles
**24**	Class coumarins and derivatives	24.1 Subclass hydroxycoumarins<br>24.2 Subclass pyranocoumarins<br>24.3 Subclass coumarin glycosides
**25**	Class pteridines and derivatives	25.1 Subclass pterins and derivatives
**26**	Class imidazopyrimidines	26.1 Subclass purines and purine derivatives
**27**	Class glycerophospholipids	27.1 Subclass glycerophosphoethanolamines<br>27.2 Subclass glycerophosphocholines<br>27.3 Subclass glycerophosphates
**28**	Class isoflavonoids	28.1 Subclass furanoisoflavonoids
**29**	Class naphthalenes	29.1 Subclass naphthols and derivatives<br>29.2 Subclass naphthoquinones
**30**	Class linear 1,3-diarylpropanoids	30.1 Subclass chalcones and dihydrochalcones
**31**	Class lactones	31.1 Subclass gamma butyrolactones

**32**	Class azoles	32.1 Subclass thiazoles<br><br>32.2 Subclass pyrazoles<br><br>32.3 Subclass imidazoles
